# Targeting Nitric Oxide with Natural Derived Compounds as a Therapeutic Strategy in Vascular Diseases

**DOI:** 10.1155/2016/7364138

**Published:** 2016-08-29

**Authors:** Maurizio Forte, Valeria Conti, Antonio Damato, Mariateresa Ambrosio, Annibale A. Puca, Sebastiano Sciarretta, Giacomo Frati, Carmine Vecchione, Albino Carrizzo

**Affiliations:** ^1^IRCCS Neuromed, Vascular Physiopathology Unit, Pozzilli, Italy; ^2^Università degli Studi di Salerno, Medicine, Surgery and Dentistry, Baronissi, Italy; ^3^IRCCS Multimedica, Milan, Italy; ^4^Department of Medico-Surgical Sciences and Biotechnologies, Sapienza University of Rome, Rome, Italy

## Abstract

Within the family of endogenous gasotransmitters, nitric oxide (NO) is the smallest gaseous intercellular messenger involved in the modulation of several processes, such as blood flow and platelet aggregation control, essential to maintain vascular homeostasis. NO is produced by nitric oxide synthases (NOS) and its effects are mediated by cGMP-dependent or cGMP-independent mechanisms. Growing evidence suggests a crosstalk between the NO signaling and the occurrence of oxidative stress in the onset and progression of vascular diseases, such as hypertension, heart failure, ischemia, and stroke. For these reasons, NO is considered as an emerging molecular target for developing therapeutic strategies for cardio- and cerebrovascular pathologies. Several natural derived compounds, such as polyphenols, are now proposed as modulators of NO-mediated pathways. The aim of this review is to highlight the experimental evidence on the involvement of nitric oxide in vascular homeostasis focusing on the therapeutic potential of targeting NO with some natural compounds in patients with vascular diseases.

## 1. Introduction

**Figure 1:**
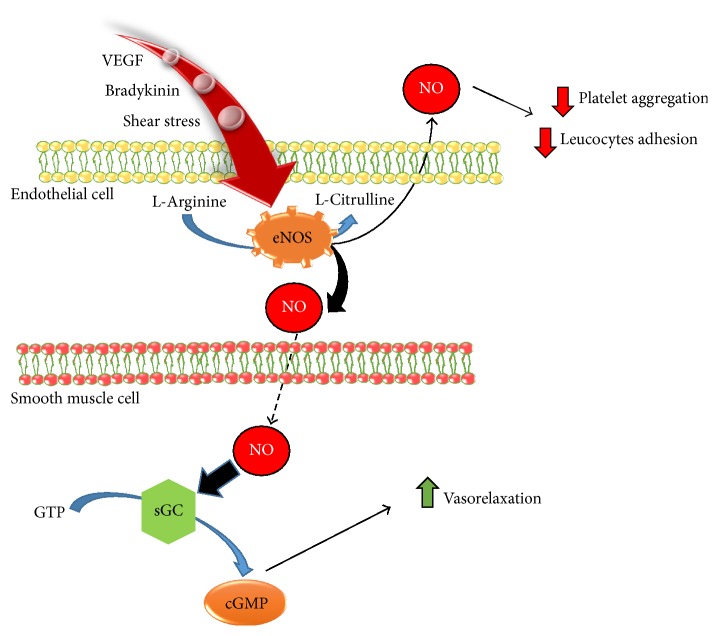
Nitric oxide generation: several stimuli induce eNOS activation and NO production in endothelial cells. NO diffusion in smooth muscle cells is responsible for cGMP generation and vasorelaxation.

Since 1992, when nitric oxide (NO) was nominated “molecule of the year” [1, 2], it continues to attract the interest of the scientific community. NO is the smallest gasotransmitter, recognized as an ubiquitous intercellular messenger; it is produced by three isoforms of NO synthases (NOS): endothelial NOS (eNOS) [3], neuronal NOS (nNOS) [4], and inducible NOS (iNOS) [5] and mitochondrial NOS (mtNOS) [6]. All NOS isozymes utilize L-arginine and oxygen and the reduced form of nicotinamide-adenine-dinucleotide phosphate (NADPH) as substrates and 6*R*-5,6,7,8-tetrahydro-L-biopterin (BH_4_) as essential cofactor to generate NO and L-citrulline [7, 8]. Then, the main downstream signaling pathway carried out by the NO is the activation of soluble guanylyl cyclase (sGC), which in turn generates cyclic guanosine monophosphate (cGMP) [9] (Figure 1).

In the vascular system NO modulates blood flow [10], vascular tone [11], and platelet aggregation [12] exerting antihypertensive, antithrombotic, and atherosclerotic effects. It is also involved in the stimulation of the endothelial progenitor cells (EPCs) and proliferation of the smooth muscle cells (SMCs) [13]. Therefore, an impairment in the NO signaling is associated with the onset and perpetuation of the main clinical condition associated to cardiovascular diseases (CVDs) including endothelial dysfunction [14].

Given this premise, it is reasonable to consider NO as a therapeutic target for CVDs. Indeed, several approaches have been proposed to modulate NO pathways while preserving its physiological role [15]. From one side, the strategy consists in enhancing NO bioavailability, principally acting on NOS cofactors or avoiding NO breakdown; from the other side, different drugs act on the NO downstream signaling targets [16].

Data from epidemiological studies have suggested the existence of a relationship between physical exercise and/or specific diets with a reduction of CVDs prevalence and incidence [17–20]. In addition, clinical trials and experiments in animal models have indicated NO as the main mediator of the beneficial effects of certain natural derived compounds, such as the polyphenols [17, 21].

In the present review, we discuss the biochemistry and pathophysiology of signaling pathways of NO focusing our attention on the experimental data showing that some natural derived compounds could be effective in the prevention and possibly treatment of CVDs.

## 2. Molecular Pathways of NO

Among the isoforms of NOS, eNOS represents the main source for the NO production in the vasculature. It is predominantly expressed in the endothelium but it has been also detected in kidney, human placenta, cardiomyocytes, platelets, and some neurons [22]. Several endogenous agonists, such as acetylcholine, bradykinin, and vascular endothelial growth factor (VEGF), as well as the shear stress induced by the blood flow, have been reported to activate eNOS [23]. Several studies have demonstrated that the phosphoinositide 3-kinase- (PI3K-) AKT pathway is mainly responsible for eNOS phosphorylation at Ser1177 especially in response to shear stress and VEGF [24–26]. Moreover, caveolin-1, the main component of the caveolae plasma membranes, has been reported as a negative regulator of eNOS [27, 28]. Another mechanism involved in the production of eNOS-derived NO is the activation of the *β*-adrenoreceptors [29] in response to the increase of catecholamines that are expressed at high levels in condition of oxidative stress associated with endothelial dysfunction [30, 31].

Neuronal NOS (nNOS) is expressed in specific neurons of the central nervous systems (CNS), as well as in the peripheral nervous systems (PNS) and in perivascular nerve fibers [32]. As in the case of eNOS, nNOS is responsible for the constitutively production of NO [33]. The inducible NOS (iNOS) is normally inactive in the vasculature [34], but its expression and activity can be induced in many cell types under oxidative and inflammatory stimuli; as a matter of fact several cytokines have been detected in the endothelium, in the media, and in the adventitia of blood vessels, as well as in neuronal cells and hepatocytes. Moreover, it is well known that NO produced by iNOS participates to the response of the immune system in killing bacteria and other exogenous compounds [35]. Several studies show the presence of a new isoform of eNOS enzyme in mitochondria (mtNOS) [36, 37]. This fourth isoform, the mtNOS, is responsible of the NO production in the mitochondria. It has been demonstrated that the NO-synthesizing capacity of mtNOS is higher than that derived from the combined activity of the all other NOS isoforms [38]. Moreover, recent findings suggest that an excessive stimulation of mtNOS leads to mitochondrial dysfunctions, which contribute to metabolic syndromes [39].

**Figure 2:**
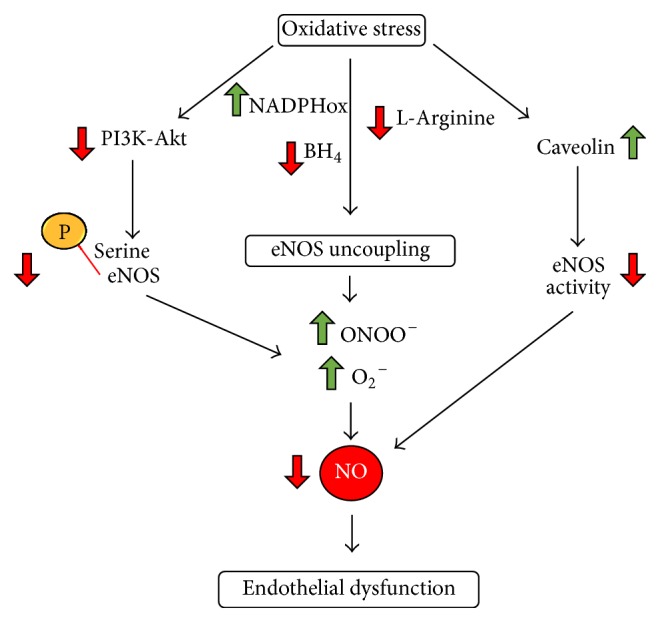
Decreased bioavailability of NO: oxidative stress is the cause of endothelial dysfunction, the common feature of CVDs; eNOS decreased activity due to different molecular pathways reduces NO production (see text for details).

**Figure 3:**
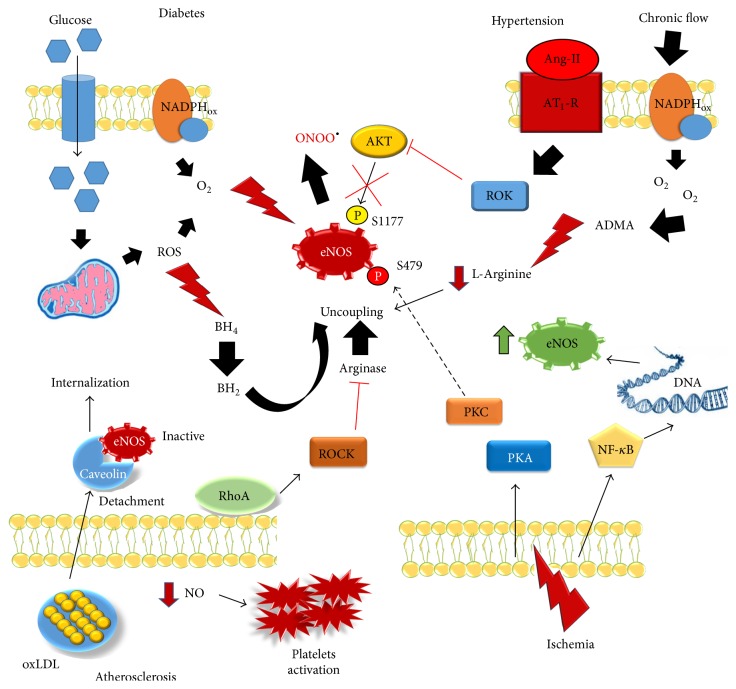
eNOS alteration, a common mechanism in different vascular diseases. The figure summarizes the main mechanisms of eNOS dysfunction promoted in the main cardiovascular diseases (see text for details).

All NOS proteins are homodimers that transfer electrons from NADPH to the haem in the oxygenase domain where there are also binding sites for BH_4_, oxygen, and L-arginine; at the haem site, the electrons are used to reduced O_2_ and to oxidize L-arginine to L-citrulline and NO. Importantly, when oxidative stress increases, eNOS can lose its physiological properties in a process termed “eNOS uncoupling” [22, 40] (Figure 2). In such condition, NO reacts with superoxide O_2_^−^, leading to formation of peroxynitrite (ONOO^−^), potent inducers of cell death, and eNOS produces reactive oxygen species (ROS), mainly O_2_^−^, rather than NO [41]. Therefore, eNOS uncoupling not only leads to decreasing NO bioavailability, but contributes to enhancing the preexisting oxidative stress [42]. Different mechanisms have been suggested to explain eNOS uncoupling; among these, the oxidation of BH_4_ to the inactive form BH_3_^−^ by O_2_^−^ and ONOO^−^ together with depletion of L-arginine plays a prominent role [4]. In particular, the decrease of L-arginine is caused by the upregulation of arginase isoforms (Arg I and Arg II) expression and activity. As we will discuss in the next sections, oxidative stress associated to eNOS uncoupling and the changing of the eNOS phosphorylation status (summarized in Figure 3) are characteristics of clinical conditions commonly associated to CVDs, such as diabetes mellitus, hypertension, atherosclerosis, and cerebral ischemia [22, 43].

### 2.1. Posttranslational Modifications of NOS

NOS enzymes are regulated by multiple interdependent mechanisms and signaling pathways, which can be calcium-dependent and/or calcium-independent. In particular, it has been demonstrated that the activity of eNOS is regulated by the increase of the cytosolic Ca^2+^ in endothelial cells, which leads to the activation of calmodulin that in turn binds eNOS, thus facilitating its function [23, 44]. Besides the increase of intracellular calcium, eNOS activity depends also on its phosphorylation status. In particular, It has been suggested that the phosphorylation of NOS isoforms at Tyr81 and Tyr657 represents a mechanism necessary to modulate the NO production above all during shear stress [45, 46].

Indeed, the phosphorylation is the major and most studied posttranslational modifications influencing the eNOS activity. Noteworthy, while the phosphorylation of serine at positions 617, 635, and 1179/1177 results in the activation of the eNOS, the same change at Ser116 and Thr497 reduces its function.

Also acetylation of the eNOS influences its activity and, in general, acetylation/deacetylation balance represents a crucial homeostatic mechanism mediating the response to metabolic changes in the cell [47].

Other important posttranslational changes are acylation, nitrosylation, glycosylation, and glutathionylation. All of them are necessary and often interconnected in controlling the subcellular localization and/or activity of the eNOS and thus the NO bioavailability in response to a variety of physiologic and pathophysiologic signals [48].

## 3. Physiopathological Role of NO in the Vascular System

The role of NO in the maintenance of vascular homeostasis is well defined and it depends on both eNOS distribution pattern and NO production rate. Perturbation of NO signaling pathways represents one of the major determinants of endothelial dysfunction, which is characterized by the reduction of the NO bioavailability and oxidative stress increase with the resulting impairment of the endothelium-dependent vasodilation [49, 50].

The NO synthesized by eNOS diffuses from endothelial cells into the underlying SMCs in which it stimulates sGC, thus generating cGMP, which in turn activates downstream protein kinases. Protein kinases predominantly act on myosin light chain phosphatase, the enzyme that dephosphorylates myosin light chains and leads to smooth muscle relaxation and vasodilatation. Moreover, NO may diffuse also in the blood flow where it inhibits several processes normally impaired during thrombotic and atherosclerotic events including platelet aggregation and leukocyte adhesion and migration into vascular wall [51].

Interestingly, over the well-known involvement of NO in the main cardio- and cerebrovascular diseases, other minor vascular forms of vascular diseases had been associated with impairment of the NO signaling. In this regard, it has been widely recognized that NO plays a key role in the physiology of penile erection eliciting its effect on guanylate cyclase leading to the production of cGMP. About this mechanism, the impairment of NO activity is similar to that observed in other forms of vascular diseases or in patients with cardiovascular risk factors (e.g., dyslipidemia, diabetes, and hypertension) [52]. Another form of vascular alteration in which changes in NO production and bioavailability have been reported is represented by varicose vein disease [53]. Furthermore, recent studies have found a link between endothelial dysfunction and NO alterations in venous valve dysfunction [53]. In particular, processes associated with varicose vein disease are increased destruction of collagen and matrix proteins triggered by endothelial dysfunction, which in turn is characterized by loss of NO bioavailability and increase of inflammation and ROS build-up [54]. Based on these findings, it is imperative to identify a new therapeutic strategy aiming at stimulating NO production and preventing the reduction of its bioavailability.

### 3.1. NO in Ischemia and Heart Failure

Many studies in animal models have documented the existence of a link between NO pathway impairment and CVDs. Kuhlencordt et al. showed that atherosclerosis, aortic aneurysm formation, and ischemic heart diseases can be accelerated as result of a chronic deficiency of eNOS [55]. The authors compared the atherosclerotic lesions occurring in two different knockout (KO) animal models, apolipoprotein E (apoE)/eNOS-double knockout (DKO) and apoE-KO, demonstrating that a genetic deficiency of eNOS significantly increased atherosclerosis in the apoE-KO mouse model. Of note, the location of the lesions, occurring mainly in the areas with disturbed flow, was similar in both KO models; therefore, the authors' conclusion was that the absence of eNOS did not determine the site of lesion formation in the aorta but appeared to accelerate its development. In addition, the ApoE/eNOS-DKO animals showed a more marked increase in blood pressure, comparable to that of eNOS-KO mice, indicating that eNOS deficiency could reflect different degrees of endothelial dysfunction. These findings are very important because they suggested eNOS deficiency/endothelial dysfunction as a possible molecular mechanism linking hypertension to atherosclerosis [55].

Actually, Huang et al. have already demonstrated that in mice lacking the gene encoding eNOS the acetylcholine-induced relaxation was absent and the eNOS mutant mice had elevated blood pressure and developed hypertension [56].

Anti-ischemic actions of NO were also demonstrated by using of a transgenic (TG) mice model with cardiac specific overexpression of iNOS. After ischemia induced by coronary occlusion followed by 24 hours of reperfusion, the TG mice had a smaller infarct size compared to wild type. In addition, iNOS overexpression was able to attenuate the ROSs generation associated with reperfusion injury, in fact, the quantity of the ROSs trapped from reperfused hearts was lower in iNOS-TG than in wild type mice [57]. In another study performed in a model of eNOS-TG mice, it was demonstrated that a cardiomyocyte-specific overexpression of eNOS improved left ventricular performance and reduced compensatory hypertrophy after myocardial infarction (MI). Importantly, eNOS cardiac overexpression attenuated also a post-MI remodeling by reducing fibrosis in the noninfarcted area of the myocardium [58]. The beneficial role of the eNOS-derived NO has been demonstrated also in congestive heart failure (HF) in the study by Jones et al. in which the authors, by using a mouse model of infarct-induced HF, showed that eNOS overexpression enhanced animal survival, inhibited pulmonary edema, and improved cardiac function but did not attenuate the cardiac hypertrophy or improve cardiac contractility [59]. Moreover, it has been reported that the mitochondrial production of NO by mtNOS is reduced during ischemia because there is a lack of the O_2_, necessary to generate the NO [36].

These findings demonstrated that strategies aimed at increasing NO bioavailability in the heart might be useful to counteract the structural and functional damage induced by myocardial ischemia.

### 3.2. NO in Diabetes and Atherosclerosis

NO production is reduced in diabetes mellitus and atherosclerosis, well-known risk factors for CVDs. In obese mouse model, eNOS activity was reported to be reduced by an enhanced phosphorylation at threonine 495 via PKC [60]. Similarly, Kashiwagi et al. showed that the lack of phosphorylation at serine 1176 residue was correlated with the development of obesity and insulin resistance in a mouse model [61].

Uncoupling eNOS also concurs to develop diabetes mellitus and, as mentioned above, both oxidation of BH_4_ and depletion of L-arginine are the cause of such phenomenon. In addition, BH_4_ was shown to be oxidized in diabetic mouse models, by a mechanism involving the activation of NADPH oxidases through PKC [62]. Similarly, in diabetic hypertensive rats, Alp et al. showed low levels of BH_4_ and decreasing in NO production [63]. Heitzer and colleagues demonstrated that a supplementation of BH_4_ improved endothelium-dependent vasodilation in patients with type II diabetes but not in control subjects. Of note, such beneficial effect was completely blocked by N(G)-monomethyl-L-arginine, a well-known inhibitor of NOS, suggesting that it was dependent on the NO production increase [64].

Also L-arginine deficiency has been reported in diabetic rats with a concomitant increase of the expression and activity of arginases, particularly, arginase, which has been recognized as the isoform responsible for eNOS uncoupling in diabetes [65]. In this regard, diabetic mice deficient of arginase I exhibited less endothelial dysfunction compared to wild type mice [66]. Notably, in the same way, in coronary arterioles of diabetic patients, arginase I was shown to contribute to the reduction of vasodilatation [67], and in plasma of patients with type II diabetes, arginase activity was reported to be elevated [68].

Interesting data in both humans and animal model have remarked the involvement of NO metabolism in the atherosclerosis. For example, depletion of BH_4_ has been demonstrated in hypercholesterolemic patients [69] and high level of superoxide anions produced by uncoupled eNOS and increased formation of aortic atherosclerotic plaque with the concomitant deficiency of BH_4_ were found in ApoE-KO mice where there were also observed an increased arginase II expression and activity [70]. Similarly, in human endothelial cells exposed to thrombin, Yang et al. found an enhancement of the arginase enzymatic activity [71]. In ApoE-KO mice, Ming et al. demonstrated that the small G protein RhoA and its effector ROCK play a role in the regulation of arginases activity involved in atherosclerotic process [72]. Moreover, posttranslational modifications of the eNOS have been shown to play a crucial role during atherosclerosis and diabetes [73]. Importantly, recent investigations have highlighted that phosphorylation and acetylation of the eNOS might concur to mediate the beneficial effects of some drugs. In this regard, Romero et al. have investigated the effects of BM-573, a compound that combines thromboxane synthase inhibition and thromboxane receptor antagonism, on endothelial dependent relaxation during early stage of atherosclerosis in apoE-KO mouse model. The authors demonstrated that BM-573 was able to ameliorate endothelial dysfunction by reducing oxidative stress and improving the NO bioavailability by increasing the eNOS phosphorylation [74]. Moreover, it has been demonstrated that lysine acetylation of the eNOS mainly contributes to the well-known atherothrombotic effects of low-dose acetylsalicylic acid [75].

The eNOS posttranslational modifications are necessary also in mediating the antidiabetic effects of several therapeutic interventions. For example, a diet supplementation with l-arginine and sepiapterin along with salsalate has been proved to increase the eNOS phosphorylation and improved vasorelaxation of thoracic/abdominal aorta in type-1 diabetic mice [76]. Furthermore, Ding et al. showed that cardiac overexpression of SIRT1, a NAD+-dependent deacetylases, reduced diabetes-exacerbated myocardial ischemia reperfusion injury and oxidative stress in diabetic rats via eNOS activation and that such effect was mediated by increase of the eNOS phosphorylation and reduction of the eNOS acetylation [77]. It has been also showed that the eNOS phosphorylation might be also important in mediating the beneficial effects of metformin and thiazolidinediones into microvasculature. In this regard, Ghosh et al. demonstrated that a brief 3 h exposure to metformin induced changes in eNOS signaling in mouse microvascular endothelial cells by reducing the ratio of phosphorylated (p-eNOS)/eNOS, but not the expression of total eNOS [78].

Xu et al. investigated the effects of ciglitazone in rat microvascular endothelial cells, finding that such antidiabetic drug was able to reverse the decrease of eNOS levels in the cells stressed with oxidized LDL thus improving the NO bioavailability [79].

### 3.3. NO and Hypertension

A decreased NO bioavailability is one of the mechanisms involved in the pathogenesis of hypertension. Indeed, the phosphorylation of eNOS at threonine 495 residue was shown to be enhanced in angiotensin II- (Ang II-) induced hypertensive rats [80]. Landmesser et al. showed an increase of BH_4_ oxidation caused by the activation of the p47phox subunit of NADPH oxidase in a model of salt-induced hypertension rat [42]. A similar enhanced expression of NAPDH oxidase has been also shown in spontaneously [22] and in angiotensin II-induced hypertensive rats [81]. In addition, an oral administration of BH_4_ was shown to suppress the hypertension in spontaneously hypertensive rats thanks to the reduction of ONOO^−^ and O_2_^−^ accumulation [82]. Similarly, a supplementation of BH_4_ increased acetylcholine-dependent endothelium vasodilatation in hypertensive patients to the level of normal control subjects [83].

Besides the oxidation of BH_4_, the depletion of L-arginine could contribute to hypertension causing eNOS/NO impairment. Indeed, in spontaneously hypertensive rats, as well as in the aorta of mineralocorticoid and salt-induced hypertensive rats, the expression/activity of arginases was found to be enhanced [84–86]. Moreover, angiotensin II, via stimulation of AT_1_, receptor is reported to be a molecular pathway responsible for the increased expression/activity of arginases in hypertension. In particular, in arginases knockout mice Shatanawi et al. showed that the p38 MAPK is the downstream effectors of AT_1_, leading to endothelial dysfunction [87]. Intravenous administration of L-arginine produces a vasodilatory effect by increasing the NO production in hypertensive individuals [88], as well as the arginase inhibitor* N-*(omega)-hydroxy-nor-l-arginine prevents the hypertension, lowering the blood pressure in a hypertensive rat model [89, 90].

### 3.4. NO and Cerebrovascular Diseases

Several experimental evidences have underlined the protective role of eNOS/NO pathways in neuronal injury after cerebral ischemia as well as in the prevention of stroke and severe subarachnoid hemorrhage (SAH) [91, 92].

In physiological conditions, eNOS-derived NO is the main molecule responsible for the control of the cerebral blood flow (CBF). In this regard, it has been shown that ischemic injury increases eNOS activity and NO availability, which in turn leads to the improvements of the CBF and to decreasing neuronal injury [93]. Osuka et al. [94], in rat cerebral models of ischemia, found increased level of phosphorylation at eNOS Ser1177 residue in microvessels, with temporary expression of VEGF. Similarly, in eNOS knockout mice, after middle cerebral artery (MCA) occlusion, Huang et al. demonstrated an enlargement of infarct size and showed that systemic administration of nitro-L-arginine prevented brain damage [43]. Moreover, thrombotic cerebral infarctions have been found in eNOS^+/−^ mice after three–six months of age [95]. Other authors underlined the importance of NO in the angiogenesis and neurogenesis occurring after cerebral stroke; for example, neovascularization after stroke was found to be impaired in eNOS deficient mice, indicating that endothelial NO mediates this effect [96].

## 4. Main Modulators of NO Pathways

Several therapeutic strategies have been proposed to ameliorate the NO homeostasis. Currently, the best strategy is based on the drugs administration in order to activate downstream effectors of eNOS/NO from one side and to reduce eNOS uncoupling [16], improving BH_4_ and L-arginine bioavailability and regulating post-translational modifications of eNOS, from the other side. Nevertheless, it is important to remark that a helpful strategy for the prevention and attenuation of CVDs is to make a good lifestyle and, in this context, physical exercise and specific diets such as diet rich in polyphenols have been suggested to improve the NO pathways.

The inhibition of the renin-angiotensin-aldosterone system is widely recognized as an effective therapy in CVDs [97]. In animal models, angiotensin-converting enzyme inhibitors (ACE-I) and AT1 receptor blockers (ARBs) are able to reduce eNOS uncoupling, while restoring BH_4_ bioavailability [98], and to protect against cerebral ischemia via upregulation of the eNOS in middle cerebral artery [99] and cerebral infarct size via eNOS activation [100].

The renin-angiotensin system blockers exert also NO-dependent antithrombotic effects. In this regard, Kucharewicz et al. demonstrated that angiotensin 1–7, a component of the renin-angiotensin system, caused an increased production of NO, which contributes to reduction of thrombosis in rats [101].

Also the cholesterol-lowering drugs, the statins, improve endothelial functions by enhancing the NO bioavailability thanks to their antioxidant, anti-inflammatory, and antiatherosclerotic properties [102, 103]. For example, in hypercholesterolemic patients treated with fluvastatin, John et al. demonstrated an improvement of endothelial vasodilatation through increase of the NO production [104]. Moreover, in a rat experimental model of MI, statins were found to enhance NO bioavailability by restoring mobilization of EPCs, myocardial neovascularization, and, ultimately, increasing survival [42] and statins were also showed to decrease eNOS uncoupling through a reduction of vascular O_2_^∙−^ and BH_4_ oxidation [105].

Another way to ameliorate endothelial homeostasis is the activation of the *β*-adrenoreceptor subtype 3 (*β*_3_), which leads to eNOS activation and thus to the NO generation by increasing the levels of cAMP and Ca^2+^ [30, 31, 106]. Nebivolol, a third-generation *β*-adrenoreceptor blocker, is a promising drug able to improve NO pathways thanks to its ability to antagonize *β*_1_ and to activate *β*_3_ receptors. Maffei et al. [107] showed that nebivolol induced endothelial NO production in both conductance and resistance rats arteries in a calcium-dependent manner. In another study, the same authors measured in mice the heart production of the NO consequent to the stimulation of *β*_3_ receptor and iNOS increased activity, thus indicating nebivolol as therapeutic strategy for hypertension and heart failure [108].

Another aspect that deserves attention is the link between adrenergic pathway, NO bioavailability, and oxidative stress and, in this context, the beneficial effects of the nebivolol are attributable to its well-recognized antioxidant properties, which are considered an additional factor for increasing the NO bioavailability. For example, nebivolol and atenolol (a second-generation *β*-blockers) similarly reduced blood pressure values in hypertensive patients, but oxidative stress markers, such as LDL hydroperoxides, 8-isoprostanes, and ox-LDL were significantly improved only in patients treated with nebivolol [109, 110].

Moreover, in hypertensive patients, Okamoto et al. demonstrated that nebivolol lowered blood pressure [111], while in elderly patients with heart failure it was shown to reduce mortality and morbidity [112]. Interestingly, Falciani et al. highlighted also the role of nebivolol in inhibiting platelet aggregation by increasing L-arginine/NO, remarking also an antithrombotic effect of this *β*-blocker [113].

Nowadays, researchers are paying particular attention to the nonpharmacological strategies, including adoption of specific diet habits and exercise programs for the management of several chronic diseases.

In this context, several experimental and epidemiological findings have underlined the role of physical exercise (PE) in decreasing the oxidative stress associated with aging and in the prevention and attenuation of CVDs-associated risk factors [114–117]. It was suggested that the reduction of oxidative stress triggered by PE could be associated with the improvement of the NO function [118]. In this regard, in patients with chronic heart failure and coronary artery disease, Laurent et al. showed that water-based exercises increased NO metabolism by improving cardiorespiratory capacity and endothelial function [119]. Recently, a regular exercise was demonstrated to activate eNOS and nitrite production and to reduce oxidative stress in spontaneously hypertensive rats [120]. PE was also suggested to have a cardioprotective effects; in ischemic rats, high-intensity interval training increased NO metabolites levels and reduced myocardial infarction [121].

Different molecular mechanisms, such as phosphorylation status and transcription rate of eNOS, have been proposed to explain the effects of PE on the NO production. For example, in rats subjected to acute and chronic aerobic training eNOS mRNA levels were found to be upregulated [122]. Other authors underlined the role of *β*_3_ adrenoreceptor in mediating the effects of PE on the NO production; in particular, Calvert et al. demonstrated that exercise could improve the cardiac function in ischemic rats via *β*_3_ adrenoreceptor by increasing the eNOS phosphorylation [123].

Another molecular mechanism is represented by the NO-dependent changes in the vascular redox state and oxidative stress even if the beneficial role of the NO in this context could be complex to elucidate. In this regard, Farah et al. suggested that certain level of eNOS uncoupling could be required for exercise-induced myocardial cardioprotection during ischemia reperfusion. In particular, in such study, it was showed that eNOS uncoupling was associated with the improved myocardial antioxidant capacity that prevented excessive NO synthesis limiting the reaction between NO and O_2_^∙−^ to form peroxynitrites [124].

## 5. Crosstalk between NO and the Other Gasotransmitters

Besides the NO, other two gaseous molecules, hydrogen sulfide (H_2_S) and carbon monoxide (CO), have been recognized as “gasotransmitters” [125]. Much like their predecessor NO, H_2_S and CO have been historically considered as highly toxic and harmful agents; afterward, many investigations have showed that they not only play various physiological roles but could be effective against a number of diseases, including CVDs [126].

Indeed, also CO and H_2_S mediate muscle relaxation and vasodilatation, the first, as well as the NO, through activation of GMP and consequent elevation of cGMP levels and the second through a cGMP-independent mechanism [127, 128].

Compelling evidence has demonstrated that each member of this triad of gasotransmitter can influence each other. For example, the inhibition of the NO synthesis might increase the CO production [129], while low-dose CO has been showed to decrease the eNOS mRNA expression [130].

Recently, particular attention is paid to the role of crosstalk existing among the gasotransmitters in determining cytoprotective effects in the heart and vessels. It was demonstrated that NO, CO, and H_2_S act in concert to preserve the cardiovascular homeostasis thanks for instance to their antioxidant and anti-inflammatory properties [131].

Noteworthy, the gaseous nature of these compounds makes them attractive candidates for the treatment of several pathological conditions, especially ischemia reperfusion injury. In this regard, H_2_S have been shown to stimulate vascular remodeling after ischemia in mice by enhancing the NO production [132]. Donnarumma et al. have recently investigated in murine and swine models of ischemia reperfusion the effects of an oral administration of zofenopril, an ACE-I containing a sulfhydrylic group. The authors found that zofenopril reduced myocardial infarct size in both animal models and preserved blood flow in swines and such effects were associated with an elevation of the H_2_S and NO plasmatic levels [133].

Importantly, there are conflicting evidences on the anti-ischemic effects of the ACE-I and some studies have revealed such effects only for sulfhydryl-containing agents [134]. Moreover, it was demonstrated that an early treatment of the acute myocardial infarction with zofenopril is able to reduce morbidity and mortality any more than ramipril, dicarboxylate-containing ACE-I [135].

Therefore, the understanding of the mechanisms involved in the cytoprotective effects of all gasotransmitters either individually or together is necessary to fully exploit their therapeutic potential.

## 6. Natural Derived Compounds and NO 

Growing evidence leads to considering a healthy dieting regimen as an helpful strategy to reduce CVDs-associated risk factors acting, as well as the aforementioned drugs, via modulation of the NO pathways.

The Mediterranean diet, rich in fruits and vegetables and based on high consumption of red wine, was associated with a good prognosis in patients with CVDs [136–139]. In particular, in subjects who usually consume large amounts of fruits, vegetables, red wine, tea, chocolate, and nuts, a significant improvement of endothelial function has been reported, which in turn contributes to reduction of blood pressure, atherosclerosis, and cardiovascular mortality [140, 141]. Interestingly, the beneficial properties of the red wine were recognized as the solution of the “French paradox,” a term used to describe the observation that the French population had a low incidence of CVDs, despite a diet predominantly characterized by a high consumption of wine and saturated fat food [142]. The protective effects against CVDs have been attributed, at least in part, to the high content in these specific foods of polyphenols, a class of chemicals characterized by the presence of phenol units in their chemical structure [143].

### 6.1. Classification and Source of Polyphenols

#### 6.1.1. Flavonoids

Flavonoids represent a large group of polyphenols, characterized by two benzene rings linked via a heterocyclic pyran ring. The latter gives reason of the differences between the various classes of flavonoids. According to their chemical structure, the flavonoids can be subdivided in (i) flavones, such as apigenin (bilberry, raspberry, strawberry, plum, cherry, blackberry, red pepper, and tomato skin) [144], (ii) flavonols that include quercitin (red onions, tea, wine, apples, cranberries, buckwheat, and beans) [145], (iii) isoflavones, including genistein (soy, legumes) and coumestrol (soy, red clover), also known as phytoestrogens [146], and (iv) flavanols that include catechins and epicatechin (tea, apple juice, wine, and cocoa) [147]. Interestingly, these compounds have been found also in medical plants, such as* Aloe vera* [148] and* Cannabis sativa* [149]. Flavonoids have been demonstrated to exert a plethora of beneficial effects both* in vivo* and* in vitro* and to regulate specific molecular pathways and target several genes [150, 151]. In particular, the best characterized biological property for all flavonoids, as well as for polyphenols in general, is their ability to act as antioxidants, inhibiting ROS accumulation, acting either by scavenging ROS or inhibiting enzymes involved in the ROS production or by enhancing the natural antioxidant defenses [152]. Moreover, several studies have shown and are underlining anticancer activities of the flavonoids. For example, quercitin has been shown to inhibit cell proliferation in several human cells, such as lymphoid, colon, ovarian, and gastric cells, through modulation of several genes involved in cancer progression [153]. Moreover, genistein has been recently proposed as a chemopreventive agent especially against prostate cancer, thanks to several interesting results deriving from* in vitro* and epidemiological studies [154, 155].

#### 6.1.2. Stilbenoids

Stilbenoids are a class of phenolic compounds synthesized as defense agents from the plants expressing stilbene synthase. Resveratrol is the most studied stilbenoid, but more than 400 compounds have been identified; most of them are currently used in Chinese traditional medicine [156]. Generally, stilbenoids are classified on the basis of the number of the C6-C2-C6 units (monomer, dimers, trimers, tetramers, and examers). They are present abundantly in berries (grape, blueberry, bilberry, cowberry, cranberry, and strawberries) and peanuts but are also detectable in cocoa powder, dark chocolate, and white tea [157, 158]. As mentioned for the flavonoids, several researches have suggested a role of stilbenoids as anticancer, antioxidant, and antiaging agents or as positive modulators of several human degenerative diseases [159, 160]. In this regard, resveratrol has been shown to induce apoptosis in breast cancer and prostate cells, by induction of caspases, Bax proteins, and p53 [161, 162]. Of note, a natural analog of resveratrol has been documented to inhibit growth of several cancers, such as pancreatic [163] and colon [164] cancer.

#### 6.1.3. Curcuminoids

Cucrcuminoids are chemical compounds extracted from the rhizome of* Curcuma Longa *Linn. They are characterized by a linear structure (diarylheptanoid) with two phenolic groups (C6-C7-C6) and are widely used as colorants for vegetables. Curcumin and its derivates have been demonstrated to possess numerous pharmacological activities, including anti-inflammatory, antioxidant, and antitumorigenic effects. In particular, it has been reported that curcumin is able* in vitro* to downregulate the expression of cyclin D and E and to upregulate p53 and p21, which in turn contribute to arresting cell proliferation/migration and promoting apoptosis [165, 166]. Concerning its antioxidant properties, curcumin acts prevalently as superoxide radical scavenger [167].

#### 6.1.4. Phenylethanoids

Phenylethanoids are polyphenols characterized by a phenethyl alcohol structure. Typical examples of phenylethanoids are tyrosol and its derivate oleuropein, present prevalently in olive oil and olive leaf. Oleuropein is the most abundant polyphenol in olives and thus it is receiving particular attention by the scientific community because extra virgin olive oil is an essential component of Mediterranean diet. Several studies have demonstrated that the oleuropein possesses a wide range of pharmacological properties such as antiatherogenic [168], hypotensive [169], and antidiabetic [170], as well as anticancer activity and antioxidant effects [171, 172]. Moreover, also hydroxytyrosol, a metabolite of oleuropein, has been shown to possess antioxidant properties [173] as well as anti-inflammatory, antiplatelet aggregation, antiatherogenic and cardioprotective, antimicrobial, antiviral, and anticancer activities [174].

## 7. Polyphenols and the NO Signaling 

**Figure 4:**
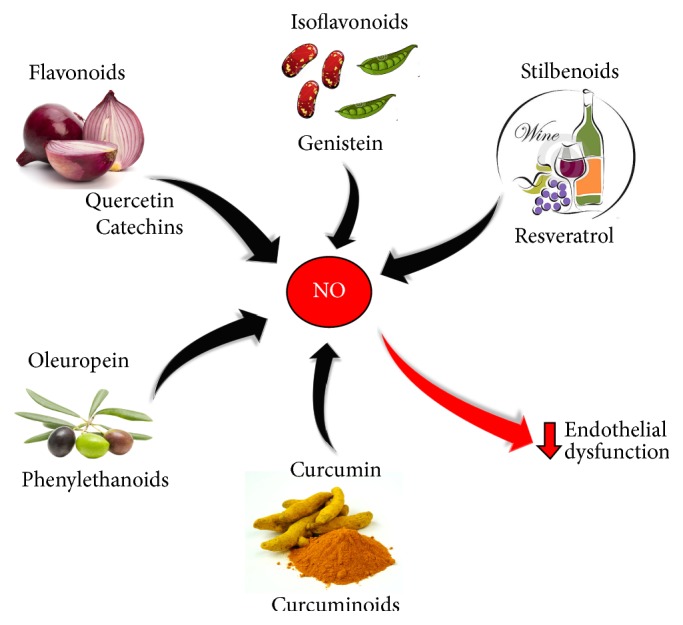
Natural derived compounds increase NO production: a diet rich in polyphenols, deriving from different sources, contributes to counteract oxidative stress and enhances NO generation, so improving the endothelial function.

Concerning the effects on vascular physiology, several data suggest that polyphenols act on the NO signaling and metabolism, improving eNOS expression and activity, as well as reducing eNOS uncoupling. Nowadays, one of the limits during the characterization of the molecular pathways activated by polyphenols is that most of the experiments have been conducted with the total extracts of food, such as wine, cocoa powder, or olive leafs extracts; therefore often it is very difficult to identify the specific compound exerting protective effects. Nevertheless, some studies measured the effects of single compounds, such as resveratrol, quercitin, or curcumin [175]. Irrespective of their source, one of the main effects exerted by the polyphenols is the NO-dependent vasodilatation (Figure 4). For example, in isolated arteries of rabbits, Karim et al. demonstrated that cocoa extracts increased levels of intracellular Ca^2+^, leading to L-arginine conversion in citrulline and to the eNOS activation [176]. Similarly, plant-derived polyphenols have been reported to induce vasodilatation of porcine coronary arteries through NO generation [177]. Moreover, in bovine endothelial cells, catechins of green tea activated eNOS by phosphorylation at Ser1179 and dephosphorylation at Thr495 in a PKA-Akt dependent manner [178, 179]. In addition, such compounds were also shown to exert protective effects in diabetic rats thanks to the reduction of oxidative stress obtained by downregulation of NADPH oxidase [180]. Interestingly, catechins were found to reduce platelet aggregation and to reverse endothelial dysfunction in patients with coronary artery disease, thus exerting antiatherosclerotic properties [181, 182]. Moreover, polyphenols of the black tea were found to enhance the activity of eNOS via p38 MAPK-dependent phosphorylation in porcine aortic endothelial cells. In fact, both pharmacological and genetic inhibition of p38 MAPK attenuated both eNOS activation and phosphorylation changes in response to these polyphenols [183].

Among plant-derived polyphenols, fruit extracts of* Camelia japonica *(CJF), a plant widely distributed in Asia and well known for its antioxidant properties [184], have been demonstrated to induce the NO production via Akt pathways in endothelial cells and to activate eNOS via phosphorylation at Ser1179. In the same study, CJF inhibited VSMCs proliferation and migration, suggesting its beneficial role in the prevention of atherosclerosis [185]. Similarly, polyphenols of the tropical plant* Terminalia* have been reported to induce a calcium-dependent activation of eNOS [186].

Interestingly, Appeldoorn et al. by using an* in vitro* screening to discover the potential effects of different polyphenols have found that quercitin, abundant in many vegetables and fruits, is one of the major stimulator of the NO production [187]. Indeed, the effects of quercitin have been extensively investigated in animal models of CVDs, especially with regard to its antihypertensive effects. For example, a reduction of blood pressure after administration of quercitin in spontaneously hypertensive rats has been showed [188], as well as in salt-hypertensive [189, 190] and NO deficient rats [191]. Recently, it has been reported that quercitin is able to ameliorate arterial erectile dysfunctions in rats via NOS regulation restoring, almost in part, the function of NO-cGMP pathway in the process of penis erection [192].

The molecular mechanism involved in the antihypertensive effect of the flavonoid quercitin was attributed to the inhibition/downregulation of NADPH oxidase. Concerning this, Perez-Vizcaino et al. demonstrated that quercitin was able to induce the lowering of blood pressure by diminishing superoxide-driven NO inactivation via downregulation of aortic p47phox, a regulatory subunit of NADPH oxidase, which is the main source of vascular superoxide [193]. These results are in accordance with others showing that quercitin decreased NADPH oxidase-mediated superoxide anion generation, as a consequence of inhibition of p47 protein subunit expression in [194].

In isolated rat aortic ring, Jin et al. found that apigenin, a polyphenol abundant in many plants, enhanced the NO bioavailability via reduction of oxidative stress. Apigenin evoked a concentration-dependent relaxation in aortas, which was specifically inhibited by L-NAME, a direct inhibitor of NOS. Of note, vasodilation occurred concomitantly with inhibition of superoxide anion and increasing of the NO levels [195]. In a similar way, curcumin has been reported to increase relaxation in porcine coronary arteries, probably thanks to mechanism involving NO, cGMP, and adrenergic *β*-receptor and, also in this case, such relaxant effect was specifically inhibited by L-NAME [196].

The involvement of caveolin-1 in polyphenols-mediated effects on the NO pathways has also been reported. Li et al. demonstrated in endothelial cells that green tea extracts downregulated the caveolin expression via activation of ERK and deactivation of p38 MAPK kinases [197]. Similarly, Vera et al. found in hypertensive rats that genistein, a soy isoflavone, was able to enhance eNOS activity via inhibition of caveolin-1 and NADPH oxidase and favoring O_2_^−^ reduction, thereby leading to decrease in blood pressure [198]. Moreover, soy isoflavones has also been demonstrated to improve the NO metabolism in carotid and cerebral rat arteries [199] as well as to enhance eNOS mRNA expression [200].

NO-mediated antihypertensive effects were also reported in rats after administration of other soy isoflavones, such as glucosyl hesperidin [201]. Yamamoto et al. found that the hypotensive effects of this natural compound were associated with reduction of oxidative stress and improvement of the NO metabolism [202]. In this regard, hesperidin was found to significantly prevent endothelial damage and leucocytes adhesion in animal models of ischemia reperfusion. Concomitantly, an increase of NO bioavailability and a reduction of inflammatory molecules which contribute to ameliorate edema and other symptoms of venous diseases have been reported [203].

Polyphenol-rich cocoa extracts have been demonstrated to reduce blood pressure in spontaneously hypertensive rats [204] and, similarly, in hypertensive patients, as well in healthy subjects, the intake of black cocoa extracts has been reported to reduce blood pressure and improve endothelial function through increase of the NO bioavailability [205–208]. Moreover, in patients with high cardiovascular risk it was showed that the administration of two different diets, one rich in polyphenols deriving from extra virgin olive oil and another rich in nuts, was shown to reduce systolic and diastolic pressure concomitantly with an increase of the NO plasma levels [209].

### 7.1. Red Wine Polyphenols and NO Pathways

Red wine is one of the main sources of the natural polyphenols. As mentioned above, epidemiological studies have suggested that the high consumption of red wine correlates with a reduction of the CVDs risk factors. The evidence corroborating vascular effects of red wine polyphenols (RWPs), as well as grape seed extracts (GSEs) and grape juice polyphenols (GJPs), is the induction of NO-dependent relaxation in isolate arteries and the activation of NO signaling pathways in endothelial cells [210–212]. Leikert et al. found that RWPs enhanced eNOS expression and release of NO in human endothelial cells [213]. In the same way, NO production and intracellular Ca^2+^ release have been shown in bovine endothelial cells treated with RWPs [214] and an increase of eNOS and Akt phosphorylation were also reported in endothelial cells exposed to GSEs [215]. Similar eNOS activation was also demonstrated in isolated arteries. For example, in porcine coronary arteries Madeira et al. showed endothelium relaxation induced by GSEs via Akt/eNOS phosphorylation [216], and also in isolated porcine coronary arteries, RWPs were found to enhance phosphorylation of eNOS at Ser1177, resulting in the increase of the NO production [217]. Interestingly, in rat femoral arteries, RWPs were shown to induce vasodilatation and to increase the NO levels in a concentration-dependent manner [218]. Moreover, RWPs were demonstrated in rat aorta to enhance NO bioavailability and to increase intracellular Ca^2+^ and cGMP concentrations [218, 219].

Several molecular mechanisms have been proposed to explain in both animal models and humans the beneficial effects of the RWPs in vascular physiology. In this regard, Bernátová et al. in hypertensive NO deficient rats showed that RWPs restored endothelial functions thanks to a reduction of blood pressure induced by increased eNOS activity in the left ventricle and aorta [220].

Similarly, in salt-induced hypertensive rats, RWPs were shown to improve vascular physiology by inhibiting NADPH oxidase [221]. The inhibition of NADPH oxidase was also reported in Ang II hypertensive rats treated with RWPs in which a reduction of superoxide anions level occurred concomitantly with restoration of the NO bioavailability [222]. RWPs have been demonstrated to exert protective effects also in animal models of ischemia and atherosclerosis. For example, in ischemic rats, RWPs were shown to reduce the angiogenic process [223], and, in hypercholesterolemic mice, Napoli et al. showed that low doses of RWPs reduced atherosclerosis by eNOS activation [224]. Interestingly, with an* in vitro* model of human atherosclerosis, Magrone et al. have reported enhanced production of the NO, after administration of red wine. The authors tested some red wines for their ability to trigger NO production from human healthy peripheral blood mononuclear cells, finding that flavonoids and resveratrol, abundant in the red wine, once absorbed at intestinal level and entered into circulation, induced monocytes to produce the NO [225].

Few clinical trials have planned with the aim to investigate the effects of a dietary regimen based on moderate consumption of wine about NO related improvement in vascular physiology in both healthy patients and patients with high risk of CVDs. For example, in healthy subjects, an oral supplementation of grape juice was found to inhibit platelet aggregation with decreased production of superoxide and enhanced NO levels [226, 227]. Moreover, besides its antithrombotic activity, red wine has also been suggested to exert cardiovascular protective effects by enhancing circulating endothelial progenitor cells thanks to a mechanism involving an increase of the NO bioavailability, as reported in studies performed in healthy individuals by Huang et al. [228]. In addition, red wine consumption has been shown to significantly decrease blood pressure and enhance plasma NO levels in hypertensive patients [229]. Interestingly, Karatzi et al. demonstrated that in smokers a consumption of red wine counterbalanced the endothelial dysfunction caused by oxidative stress induced by cigarettes smoke, in a pathway probably mediated by NO [230].

### 7.2. Resveratrol and NO Pathways

Among the RWPs, resveratrol (RSV) is one of the best characterized members. It has been used in the Indian medical herb named “Darakchasava” from about 4500 years ago and the clinical effects described in the past for “Darakchasava” are the same attributed to RSV today [231]. RSV was firstly described for its antitumorigenic properties [232]; it is present especially in grape skin and red wine, but also in peanuts, pistachios, and pine trees [233]. The interest of the scientific community for RSV derives from the observation that its administration mimics the effects of calorie restriction, a tool widely recognized to prevent the endothelial dysfunction, thereby attenuating atherosclerosis, hypertension, diabetes, and CVDs risk factors and aging-associated diseases in general [234–236]. Thanks to some experiments conducted* in vitro* in endothelial cells, RSV has been shown to regulate several target molecules, such as the NAD^+^-dependent deacetylases named sirtuins, acting at transcriptional and posttranscriptional levels [237–239].

Although the studies underlining the vascular protective effects exerted by RSV did not study the involvement of the NO signaling [157, 234], several findings, obtained in animal models of CVDs, have proposed the NO as the main downstream target mediating such effects. For example, Xia et al. demonstrated in ApoE deficient mice that RSV was able to modulate the oxidative stress responsible for atherosclerosis. From one side, NADPH oxidases were downregulated; from the other side superoxide dismutases (SOD) were upregulated. Moreover, oxidation of BH_4_ was found to be reduced, attenuating the increase of eNOS uncoupling levels [240]. Other beneficial effects were shown in many different clinical settings reinforcing the idea that RSV could be considered an optimal therapeutic strategy against CVDs. For example, in hypercholesterolemic rabbits, RSV improved endothelial function in parallel with an increase of NO plasma levels [241]. In addition, RSV has been suggested to contrast the endothelial dysfunction correlated with metabolic syndromes. In this regard, in endothelial cells RSV was demonstrated to suppress superoxide generation and to activate eNOS through phosphorylation at Ser1177 thereby increasing the NO generation [242]. In aortas of diabetic mice, RSV restored vasodilatation by enhancing eNOS activity and inhibiting the tumor necrosis factor *α*- (TNF*α*-) induced activation of NADPH oxidase [243]. In the same way, a treatment in rats with RSV has been showed to increase muscle microvascular recruitment via an NO-dependent mechanism blocked by TNF*α* [244]. Also, RSV was shown to reduce blood pressure in obese rats and to enhance the expression of eNOS via AMPK and reduction of TNF*α* in adipose tissue [245]. Similarly, in rats fed with high fructose diet, RSV decreased blood pressure via AMPK-Akt-NOS pathway [246]. Interestingly, in the myocardium of diabetic mice, RSV reduced Cav-1 expression, which in turn contributes to enhance eNOS activity [247], and the same effects on Cav-1 expression were found in hypercholesterolemic rats [248].

Furthermore, RSV was shown to protect heart from ischemic reperfusion injury. Hattori et al. demonstrated that RSV reduced infarct size in rat hearts by enhancing iNOS expression [249]. The cardioprotective effects of the RSV has also been showed in spontaneously and angiotensin Ang II-induced hypertensive rats, in which RSV contributes to the upregulation of the eNOS activity and reduction of pressure and cardiac hypertrophy [250]. Moreover, the antihypertensive effect of the RSV was also shown to be mediated by the attenuation of eNOS uncoupling via reduction of L-arginine levels and oxidative stress [251].

The antithrombotic activity of the RSV has been also reported in human platelets. Gresele et al. showed that RSV stimulated platelet NO production through inhibition of p38 MAPK, NADPH oxidases, and superoxide formation, thus decreasing peroxynitrite accumulation [252].

RSV was also shown to mobilize endothelial progenitor cells in a NO-dependent manner, thus contributing to repairing the damage occurring in vessels after ischemic injuries [253].

In the arteries of patients with hypertension and dyslipidemia, Carrizzo et al. characterized many of the downstream effectors of the RSV-dependent NO generation. The authors found an enhanced vasodilatation of arteries due to the activation of AMPK and reduction of eNOS uncoupling via increasing levels of BH_4_ and, in the same study, RSV was found to reduce vascular oxidative stress trough upregulation of manganese superoxide dismutase in a pathway mediate by nuclear factor erythroid-derived 2-like 2 [254].

Some authors have also suggested the potential therapeutic use of RSV for the prevention of stroke; for example, in rat models of stroke, RSV reduced brain damage in a NO-dependent manner [255]. Similarly, in rats subjected to focal cerebral ischemia Tsai et al. provided the evidence that RSV might enhance plasma levels of the NO and upregulate eNOS expression while it might downregulate iNOS expression and that these effects were abolished by the coadministration of selective NOS inhibitors [256].

## 8. Bioavailability of Polyphenols

Although the use of the polyphenols represents a promising tool for increasing the NO production and activity against CVDs, one of the biggest challenges for their employ in the clinical practice is to enhance their low bioavailability. In this regard, it has been shown that when orally administered, polyphenols concentration appears not to be sufficient to ensure therapeutic effects [257]. For example, the plasmatic levels of the resveratrol from dietary intake are often undetectable or very low when compared with the concentrations employed during* in vivo *and* in vitro* experiments [258]. Similarly, the pharmacological properties of curcumin are drastically restricted mainly because of its low water solubility and absorption from the gut, short half-life, and extremely poor bioavailability.

To overcome such problems, one of the best approach could be developing new pharmaceutical formulations, for example, polyphenols conjugated with cyclodextrins, or encapsulated in nanoparticles (NP), such as poly(lactic-co-glycolic acid) (PLGA) based NP or liposomes. In this regard, many of these formulations have been demonstrated to improve solubility, systemic half-life, resistance to metabolic degradation, and ultimately the bioavailability of the polyphenolic compounds in order to potentiate their biological activities [259, 260]. However, while the differences between polyphenols monoadministered or administered in encapsulated formulations have been extensively studied for what concerns the polyphenols antioxidant and anticancer properties, no experiments have been carried out on the effects of these formulations on the NO metabolism.

## 9. Conclusion

Targeting the gasotransmitter NO is becoming a new challenge in cardiovascular medicine. We here reviewed some of the experimental evidences that have indicated several natural compounds as suitable activators of the NO signaling pathways.

It is necessary to remark that for most of them the molecular mechanism, as well as the precise concentration to obtain beneficial effects, especially because of their low bioavailability remains to be determined. Nevertheless, these agents, mainly the polyphenols, doubtless possess a great therapeutic potential above all when you consider that the available drugs, although effective, did not act exclusively on the NO pathways often causing deleterious side effects. Moreover, most of the investigations on the natural compounds have involved* in vitro* studies; thus it is difficult to draw definite conclusions about their therapeutic usefulness.

Although accumulating evidence suggests that the polyphenols exert beneficial effects against vascular diseases by restoring the impairment of the NO production and/or bioavailability, much remains to be clarified. Doubtless, many gaps must be filled in understanding the complex chemistry, biochemistry, and molecular biology of such natural agents in order to introduce such NO signaling modulators in the clinical practice.

## References

[1] Lei J., Vodovotz Y., Tzeng E., Billiar T. R. (2013). Nitric oxide, a protective molecule in the cardiovascular system. *Nitric Oxide—Biology and Chemistry*.

[2] Loscalzo J., Welch G. (1995). Nitric oxide and its role in the cardiovascular system. *Progress in Cardiovascular Diseases*.

[3] Albrecht E. W. J. A., Stegeman C. A., Heeringa P., Henning R. H., van Goor H. (2003). Protective role of endothelial nitric oxide synthase. *The Journal of Pathology*.

[4] Förstermann U., Sessa W. C. (2012). Nitric oxide synthases: regulation and function. *European Heart Journal*.

[5] Alderton W. K., Angell A. D. R., Craig C. (2005). GW274150 and GW273629 are potent and highly selective inhibitors of inducible nitric oxide synthase in vitro and in vivo. *British Journal of Pharmacology*.

[6] Giulivi C., Poderoso J. J., Boveris A. (1998). Production of nitric oxide by mitochondria. *The Journal of Biological Chemistry*.

[7] Alderton W. K., Cooper C. E., Knowles R. G. (2001). Nitric oxide synthases: structure, function and inhibition. *Biochemical Journal*.

[8] Crane B. R., Arvai A. S., Ghosh D. K. (1998). Structure of nitric oxide synthase oxygenase dimer with pterin and substrate. *Science*.

[9] Murad F. (2006). Nitric oxide and cyclic GMP in cell signaling and drug development. *The New England Journal of Medicine*.

[10] Moncada S., Higgs E. A. (2006). The discovery of nitric oxide and its role in vascular biology. *British Journal of Pharmacology*.

[11] Li H., Horke S., Förstermann U. (2014). Vascular oxidative stress, nitric oxide and atherosclerosis. *Atherosclerosis*.

[12] Förstermann U. (2010). Nitric oxide and oxidative stress in vascular disease. *Pflügers Archiv*.

[13] Fleissner F., Thum T. (2011). Critical role of the nitric oxide/reactive oxygen species balance in endothelial progenitor dysfunction. *Antioxidants and Redox Signaling*.

[14] Montezano A. C., Touyz R. M. (2012). Reactive oxygen species and endothelial function—role of nitric oxide synthase uncoupling and nox family nicotinamide adenine dinucleotide phosphate oxidases. *Basic and Clinical Pharmacology and Toxicology*.

[15] Luiking Y. C., Engelen M. P. K. J., Deutz N. E. P. (2010). Regulation of nitric oxide production in health and disease. *Current Opinion in Clinical Nutrition & Metabolic Care*.

[16] Lundberg J. O., Gladwin M. T., Weitzberg E. (2015). Strategies to increase nitric oxide signalling in cardiovascular disease. *Nature Reviews Drug Discovery*.

[17] Desideri G., Kwik-Uribe C., Grassi D. (2012). Benefits in cognitive function, blood pressure, and insulin resistance through cocoa flavanol consumption in elderly subjects with mild cognitive impairment: the Cocoa, Cognition, and Aging (CoCoA) study. *Hypertension*.

[18] Schroeter H., Heiss C., Spencer J. P. E., Keen C. L., Lupton J. R., Schmitz H. H. (2010). Recommending flavanols and procyanidins for cardiovascular health: current knowledge and future needs. *Molecular Aspects of Medicine*.

[19] Francescomarino S. D., Sciartilli A., Valerio V. D., Baldassarre A. D., Gallina S. (2009). The effect of physical exercise on endothelial function. *Sports Medicine*.

[20] Moyna N. M., Thompson P. D. (2004). The effect of physical activity on endothelial function in man. *Acta Physiologica Scandinavica*.

[21] Heiss C., Keen C. L., Kelm M. (2010). Flavanols and cardiovascular disease prevention. *European Heart Journal*.

[22] Li H., Förstermann U. (2014). Pharmacological prevention of eNOS uncoupling. *Current Pharmaceutical Design*.

[23] Fleming I. (2010). Molecular mechanisms underlying the activation of eNOS. *Pflügers Archiv—European Journal of Physiology*.

[24] Soucy K. G., Ryoo S., Benjo A. (2006). Impaired shear stress-induced nitric oxide production through decreased NOS phosphorylation contributes to age-related vascular stiffness. *Journal of Applied Physiology*.

[25] Dimmeler S., Assmus B., Hermann C., Haendeler J., Zeiher A. M. (1998). Fluid shear stress stimulates phosphorylation of Akt in human endothelial cells: involvement in suppression of apoptosis. *Circulation Research*.

[26] Lee S., Chen T. T., Barber C. L. (2007). Autocrine VEGF signaling is required for vascular homeostasis. *Cell*.

[27] García-Cardeña G., Fan R., Stern D. F., Liu J., Sessa W. C. (1996). Endothelial nitric oxide synthase is regulated by tyrosine phosphorylation and interacts with caveolin-1. *The Journal of Biological Chemistry*.

[28] Feron O., Michel J. B., Sase K., Michel T. (1998). Dynamic regulation of endothelial nitric oxide synthase: complementary roles of dual acylation and caveolin interactions. *Biochemistry*.

[29] Trochu J.-N., Leblais V., Rautureau Y. (1999). Beta 3-adrenoceptor stimulation induces vasorelaxation mediated essentially by endothelium-derived nitric oxide in rat thoracic aorta. *British Journal of Pharmacology*.

[30] Dessy C., Moniotte S., Ghisdal P., Havaux X., Noirhomme P., Balligand J. L. (2004). Endothelial *β*3-adrenoceptors mediate vasorelaxation of human coronary microarteries through nitric oxide and endothelium-dependent hyperpolarization. *Circulation*.

[31] Dessy C., Saliez J., Ghisdal P. (2005). Endothelial *β*3-adrenoreceptors mediate nitric oxide-dependent vasorelaxation of coronary microvessels in response to the third-generation *β*-blocker nebivolol. *Circulation*.

[32] Korneev S. A., Park J.-H., O'Shea M. (1999). Neuronal expression of neural nitric oxide synthase (nNOS) protein is suppressed by an antisense RNA transcribed from an NOS pseudogene. *The Journal of Neuroscience*.

[33] Zhou L., Zhu D.-Y. (2009). Neuronal nitric oxide synthase: structure, subcellular localization, regulation, and clinical implications. *Nitric Oxide*.

[34] Förstermann U., Closs E. I., Pollock J. S. (1994). Nitric oxide synthase isozymes characterization, purification, molecular cloning, and functions. *Hypertension*.

[35] Wong J. M., Billiar T. R. (1995). Regulation and function of inducible nitric oxide synthase during sepsis and acute inflammation. *Advances in Pharmacology*.

[36] Lacza Z., Puskar M., Figueroa J. P., Zhang J., Rajapakse N., Busija D. W. (2001). Mitochondrial nitric oxide synthase is constitutively active and is functionally upregulated in hypoxia. *Free Radical Biology and Medicine*.

[37] Lacza Z., Pankotai E., Csordás A. (2006). Mitochondrial NO and reactive nitrogen species production: does mtNOS exist?. *Nitric Oxide*.

[38] Brookes P. S. (2004). Mitochondrial nitric oxide synthase. *Mitochondrion*.

[39] Litvinova L., Atochin D. N., Fattakhov N., Vasilenko M., Zatolokin P., Kirienkova E. (2015). Nitric oxide and mitochondria in metabolic syndrome. *Frontiers in Physiology*.

[40] Li H., Förstermann U. (2013). Uncoupling of endothelial NO synthase in atherosclerosis and vascular disease. *Current Opinion in Pharmacology*.

[41] Szabó C., Ischiropoulos H., Radi R. (2007). Peroxynitrite: biochemistry, pathophysiology and development of therapeutics. *Nature Reviews Drug Discovery*.

[42] Landmesser U., Dikalov S., Price S. R. (2003). Oxidation of tetrahydrobiopterin leads to uncoupling of endothelial cell nitric oxide synthase in hypertension. *The Journal of Clinical Investigation*.

[43] Huang Z., Huang P. L., Ma J. (1996). Enlarged infarcts in endothelial nitric oxide synthase knockout mice are attenuated by nitro-L-arginine. *Journal of Cerebral Blood Flow and Metabolism*.

[44] Fleming I., Busse R. (1999). Signal transduction of eNOS activation. *Cardiovascular Research*.

[45] Fulton D., Gratton J.-P., Sessa W. C. (2001). Post-translational control of endothelial nitric oxide synthase: why isn't calcium/calmodulin enough?. *Journal of Pharmacology and Experimental Therapeutics*.

[46] Fulton D., Gratton J.-P., McCabe T. J. (1999). Regulation of endothelium-derived nitric oxide production by the protein kinase Akt. *Nature*.

[47] Albaugh B. N., Arnold K. M., Denu J. M. (2011). KAT(ching) metabolism by the tail: insight into the links between lysine acetyltransferases and metabolism. *ChemBioChem*.

[48] Heiss E. H., Dirsch V. M. (2014). Regulation of eNOS enzyme activity by posttranslational modification. *Current Pharmaceutical Design*.

[49] Li H., Witte K., August M. (2006). Reversal of endothelial nitric oxide synthase uncoupling and up-regulation of endothelial nitric oxide synthase expression lowers blood pressure in hypertensive rats. *Journal of the American College of Cardiology*.

[50] Tousoulis D., Kampoli A.-M., Papageorgiou C. T. N., Stefanadis C. (2012). The role of nitric oxide on endothelial function. *Current Vascular Pharmacology*.

[51] Li H., Förstermann U. (2000). Nitric oxide in the pathogenesis of vascular disease. *The Journal of Pathology*.

[52] Virag R., Bouilly P., Frydman D. (1985). Is impotence an arterial disorder? A study of arterial risk factors in 440 impotent men. *The Lancet*.

[53] Guzik B., Chwała M., Matusik P. (2011). Mechanisms of increased vascular superoxide production in human varicose veins. *Polskie Archiwum Medycyny Wewnętrznej*.

[54] Yasim A., Kilinç M., Aral M. (2008). Serum concentration of procoagulant, endothelial and oxidative stress markers in early primary varicose veins. *Phlebology*.

[55] Kuhlencordt P. J., Gyurko R., Han F. (2001). Accelerated atherosclerosis, aortic aneurysm formation, and ischemic heart disease in apolipoprotein E/endothelial nitric oxide synthase double-knockout mice. *Circulation*.

[56] Huang P. L., Huang Z., Mashimo H. (1995). Hypertension in mice lacking the gene for endothelial nitric oxide synthase. *Nature*.

[57] West M. B., Rokosh G., Obal D. (2008). Cardiac myocyte-specific expression of inducible nitric oxide synthase protects against ischemia/reperfusion injury by preventing mitochondrial permeability transition. *Circulation*.

[58] Janssens S., Pokreisz P., Schoonjans L. (2004). Cardiomyocyte-specific overexpression of nitric oxide synthase 3 improves left ventricular performance and reduces compensatory hypertrophy after myocardial infarction. *Circulation Research*.

[59] Jones S. P., Greer J. J. M., van Haperen R., Duncker D. J., de Crom R., Lefer D. J. (2003). Endothelial nitric oxide synthase overexpression attenuates congestive heart failure in mice. *Proceedings of the National Academy of Sciences of the United States of America*.

[60] Taguchi K., Kobayashi T., Matsumoto T., Kamata K. (2011). Dysfunction of endothelium-dependent relaxation to insulin via PKC-mediated GRK2/Akt activation in aortas of ob/ob mice. *American Journal of Physiology—Heart and Circulatory Physiology*.

[61] Kashiwagi S., Atochin D. N., Li Q. (2013). ENOS phosphorylation on serine 1176 affects insulin sensitivity and adiposity. *Biochemical and Biophysical Research Communications*.

[62] Beral V., Bull D., Green J., Reeves G. (2007). Ovarian cancer and hormone replacement therapy in the Million Women Study. *The Lancet*.

[63] Alp N. J., Mussa S., Khoo J. (2003). Tetrahydrobiopterin-dependent preservation of nitric oxide-mediated endothelial function in diabetes by targeted transgenic GTP-cyclohydrolase I overexpression. *The Journal of Clinical Investigation*.

[64] Heitzer T., Krohn K., Albers S., Meinertz T. (2000). Tetrahydrobiopterin improves endothelium-dependent vasodilation by increasing nitric oxide activity in patients with Type II diabetes mellitus. *Diabetologia*.

[65] Romero M. J., Platt D. H., Tawfik H. E. (2008). Diabetes-induced coronary vascular dysfunction involves increased arginase activity. *Circulation Research*.

[66] Romero M., Iddings J., Platt D. (2012). Diabetes-induced vascular dysfunction involves arginase I. *American Journal of Physiology—Heart and Circulatory Physiology*.

[67] Beleznai T., Feher A., Spielvogel D., Lansman S. L., Bagi Z. (2011). Arginase 1 contributes to diminished coronary arteriolar dilation in patients with diabetes. *American Journal of Physiology—Heart and Circulatory Physiology*.

[68] Kashyap S. R., Lara A., Zhang R., Park Y. M., DeFronzo R. A. (2008). Insulin reduces plasma arginase activity in type 2 diabetic patients. *Diabetes Care*.

[69] Stroes E., Kastelein J., Cosentino F. (1997). Tetrahydrobiopterin restores endothelial function in hypercholesterolemia. *The Journal of Clinical Investigation*.

[70] Erdely A., Kepka-Lenhart D., Salmen-Muniz R. (2010). Arginase activities and global arginine bioavailability in wild-type and ApoE-deficient mice: responses to high fat and high cholesterol diets. *PLoS ONE*.

[71] Yang L., Lewis C. M., Chandrasekharan U. M., Kinney C. M., DiCorleto P. E., Kashyap V. S. (2006). Arginase activity is increased by thrombin: a mechanism for endothelial dysfunction in arterial thrombosis. *Journal of the American College of Surgeons*.

[72] Ming X.-F., Barandier C., Viswambharan H. (2004). Thrombin stimulates human endothelial arginase enzymatic activity via RhoA/ROCK pathway: implications for atherosclerotic endothelial dysfunction. *Circulation*.

[73] Kolluru G. K., Siamwala J. H., Chatterjee S. (2010). ENOS phosphorylation in health and disease. *Biochimie*.

[74] Romero M., Leon-Gomez E., Lobysheva I. (2016). Effects of BM-573 on endothelial dependent relaxation and increased blood pressure at early stages of atherosclerosis. *PLoS ONE*.

[75] Jung S.-B., Kim C.-S., Naqvi A. (2010). Histone deacetylase 3 antagonizes aspirin-stimulated endothelial nitric oxide production by reversing aspirin-induced lysine acetylation of endothelial nitric oxide synthase. *Circulation Research*.

[76] Krishnan M., Janardhanan P., Roman L. (2015). Enhancing eNOS activity with simultaneous inhibition of IKK*β* restores vascular function in Ins2Akita^+/−^ type-1 diabetic mice. *Laboratory Investigation*.

[77] Ding M., Lei J., Han H. (2015). SIRT1 protects against myocardial ischemia-reperfusion injury via activating eNOS in diabetic rats. *Cardiovascular Diabetology*.

[78] Ghosh S., Lakshmanan A. P., Hwang M. J. (2015). Metformin improves endothelial function in aortic tissue and microvascular endothelial cells subjected to diabetic hyperglycaemic conditions. *Biochemical Pharmacology*.

[79] Xu L., Wang S., Li B., Sun A., Zou Y., Ge J. (2015). A protective role of ciglitazone in ox-LDL-induced rat microvascular endothelial cells via modulating PPAR*γ*-dependent AMPK/eNOS pathway. *Journal of Cellular and Molecular Medicine*.

[80] Ramseyer V. D., Gonzalez-Vicente A., Carretero O. A., Garvin J. L. (2015). Angiotensin II-induced hypertension blunts thick ascending limb NO production by reducing no synthase 3 expression and enhancing threonine 495 phosphorylation. *American Journal of Physiology—Renal Physiology*.

[81] Mollnau H., Wendt M., Szöcs K. (2002). Effects of angiotensin II infusion on the expression and function of NAD(P)H oxidase and components of nitric oxide/cGMP signaling. *Circulation research*.

[82] Hong H.-J., Hsiao G., Cheng T.-H., Yen M.-H. (2001). Supplemention with tetrahydrobiopterin suppresses the development of hypertension in spontaneously hypertensive rats. *Hypertension*.

[83] Higashi Y., Sasaki S., Nakagawa K. (2002). Tetrahydrobiopterin enhances forearm vascular response to acetylcholine in both normotensive and hypertensive individuals. *American Journal of Hypertension*.

[84] Demougeot C., Prigent-Tessier A., Bagnost T. (2007). Time course of vascular arginase expression and activity in spontaneously hypertensive rats. *Life Sciences*.

[85] Johnson F. K., Johnson R. A., Peyton K. J., Durante W. (2005). Arginase inhibition restores arteriolar endothelial function in Dahl rats with salt-induced hypertension. *American Journal of Physiology—Regulatory Integrative and Comparative Physiology*.

[86] Rodriguez S., Richert L., Berthelot A. (2000). Increased arginase activity in aorta of mineralocorticoid-salt hypertensive rats. *Clinical and Experimental Hypertension*.

[87] Shatanawi A., Romero M. J., Iddings J. A. (2011). Angiotensin II-induced vascular endothelial dysfunction through RhoA/Rho kinase/p38 mitogen-activated protein kinase/arginase pathway. *American Journal of Physiology—Cell Physiology*.

[88] Hishikawa K., Nakaki T., Suzuki H., Kato R., Saruta T. (1993). Role of l-arginine-nitric oxide pathway in hypertension. *Journal of Hypertension*.

[89] Demougeot C., Prigent-Tessier A., Marie C., Berthelot A. (2005). Arginase inhibition reduces endothelial dysfunction and blood pressure rising in spontaneously hypertensive rats. *Journal of Hypertension*.

[90] Bagnost T., Berthelot A., Bouhaddi M. (2008). Treatment with the arginase inhibitor N*ω*-hydroxy-nor-L-arginine improves vascular function and lowers blood pressure in adult spontaneously hypertensive rat. *Journal of Hypertension*.

[91] Iadecola C. (1997). Bright and dark sides of nitric oxide in ischemic brain injury. *Trends in Neurosciences*.

[92] Dalkara T., Morikawa E., Panahian N., Moskowitz M. A. (1994). Blood flow-dependent functional recovery in a rat model of focal cerebral ischemia. *American Journal of Physiology—Heart and Circulatory Physiology*.

[93] Gajkowska B., Mossakowski M. J. (1997). Endothelial nitric oxide synthase in vascular endothelium of rat hippocampus after ischemia: evidence and significance. *Folia Neuropathologica*.

[94] Osuka K., Watanabe Y., Usuda N., Nakazawa A., Tokuda M., Yoshida J. (2004). Modification of endothelial NO synthase through protein phosphorylation after forebrain cerebral ischemia/reperfusion. *Stroke*.

[95] Morikawa E., Moskowitz M. A., Huang Z., Yoshida T., Irikura K., Dalkara T. (1994). L-arginine infusion promotes nitric oxide-dependent vasodilation, increases regional cerebral blood flow, and reduces infarction volume in the rat. *Stroke*.

[96] Cui X., Chopp M., Zacharek A., Zhang C., Roberts C., Chen J. (2009). Role of endothelial nitric oxide synthetase in arteriogenesis after stroke in mice. *Neuroscience*.

[97] Ghiadoni L., Taddei S., Virdis A. (2012). Hypertension and endothelial dysfunction: therapeutic approach. *Current Vascular Pharmacology*.

[98] Wenzel P., Schulz E., Oelze M. (2008). AT1-receptor blockade by telmisartan upregulates GTP-cyclohydrolase I and protects eNOS in diabetic rats. *Free Radical Biology and Medicine*.

[99] Liu H., Kitazato K. T., Uno M. (2008). Protective mechanisms of the angiotensin II type 1 receptor blocker candesartan against cerebral ischemia: in-vivo and in-vitro studies. *Journal of Hypertension*.

[100] Engelhorn T., Doerfler A., Heusch G., Schulz R. (2006). Reduction of cerebral infarct size by the AT1-receptor blocker candesartan, the HMG-CoA reductase inhibitor rosuvastatin and their combination. An experimental study in rats. *Neuroscience Letters*.

[101] Kucharewicz I., Pawlak R., Matys T., Pawlak D., Buczko W. (2002). Antithrombotic effect of captopril and losartan is mediated by angiotensin-(1–7). *Hypertension*.

[102] Davignon J. (2004). Beneficial cardiovascular pleiotropic effects of statins. *Circulation*.

[103] Liao J. K., Laufs U. (2005). Pleiotropic effects of statins. *Annual Review of Pharmacology and Toxicology*.

[104] John S., Schlaich M., Langenfeld M. (1998). Increased bioavailability of nitric oxide after lipid-lowering therapy in hypercholesterolemic patients: a randomized, placebo-controlled, double-blind study. *Circulation*.

[105] Antoniades C., Bakogiannis C., Leeson P. (2011). Rapid, direct effects of statin treatment on arterial redox state and nitric oxide bioavailability in human atherosclerosis via tetrahydrobiopterin- mediated endothelial nitric oxide synthase coupling. *Circulation*.

[106] Maffei A., Lembo G. (2009). Nitric oxide mechanisms of nebivolol. *Therapeutic Advances in Cardiovascular Disease*.

[107] Maffei A., Vecchione C., Aretini A. (2006). Characterization of nitric oxide release by nebivolol and its metabolites. *American Journal of Hypertension*.

[108] Maffei A., Di Pardo A., Carangi R. (2007). Nebivolol induces nitric oxide release in the heart through inducible nitric oxide synthase activation. *Hypertension*.

[109] Conti V., Russomanno G., Corbi G., Izzo V., Vecchione C., Filippelli A. (2013). Adrenoreceptors and nitric oxide in the cardiovascular system. *Frontiers in Physiology*.

[110] Mason R. P., Kubant R., Jacob R. F., Walter M. F., Boychuk B., Malinski T. (2006). Effect of nebivolol on endothelial nitric oxide and peroxynitrite release in hypertensive animals: role of antioxidant activity. *Journal of Cardiovascular Pharmacology*.

[111] Okamoto L. E., Gamboa A., Shibao C. A. (2014). Nebivolol, but not metoprolol, lowers blood pressure in nitric oxide-sensitive human hypertension. *Hypertension*.

[112] Flather M. D., Shibata M. C., Coats A. J. S. (2005). Randomized trial to determine the effect of nebivolol on mortality and cardiovascular hospital admission in elderly patients with heart failure (SENIORS). *European Heart Journal*.

[113] Falciani M., Rinaldi B., D'Agostino B. (2001). Effects of nebivolol on human platelet aggregation. *Journal of Cardiovascular Pharmacology*.

[114] Conti V., Corbi G., Russomanno G. (2012). Oxidative stress effects on endothelial cells treated with different athletes’ sera. *Medicine & Science in Sports & Exercise*.

[115] Conti V., Russomanno G., Corbi G., Filippelli A. (2012). Exercise training in aging and diseases. *Translational Medicine @ UniSa*.

[116] Conti V., Russomanno G., Corbi G. (2013). Aerobic training workload affects human endothelial cells redox homeostasis. *Medicine and Science in Sports and Exercise*.

[117] Puca A. A., Carrizzo A., Villa F. (2013). Vascular ageing: the role of oxidative stress. *The International Journal of Biochemistry & Cell Biology*.

[118] Linke A., Erbs S., Hambrecht R. (2008). Effects of exercise training upon endothelial function in patients with cardiovascular disease. *Frontiers in Bioscience*.

[119] Laurent M., Daline T., Malika B. (2009). Training-induced increase in nitric oxide metabolites in chronic heart failure and coronary artery disease: an extra benefit of water-based exercises?. *European Journal of Cardiovascular Prevention & Rehabilitation*.

[120] Kumral Z., Sener G., Ozgur S. (2016). Regular exercise alleviates renovascular hypertension-induced cardiac/endothelial dysfunction and oxidative injury in rats. *Journal of Physiology and Pharmacology*.

[121] Fallahi A., Gaeini A., Shekarfroush S., Khoshbaten A. (2015). Cardioprotective effect of high intensity interval training and nitric oxide metabolites (NO_2_
^−^, NO_3_
^−^). *Iranian Journal of Public Health*.

[122] Faulx M. D., Wright A. T., Hoit B. D. (2003). Detection of endothelial dysfunction with brachial artery ultrasound scanning. *American Heart Journal*.

[123] Calvert J. W., Condit M. E., Aragón J. P. (2011). Exercise protects against myocardial ischemia-reperfusion injury via stimulation of *β*3-adrenergic receptors and increased nitric oxide signaling: role of nitrite and nitrosothiols. *Circulation Research*.

[124] Farah C., Kleindienst A., Bolea G. (2013). Exercise-induced cardioprotection: a role for eNOS uncoupling and NO metabolites. *Basic Research in Cardiology*.

[125] Mustafa A. K., Gadalla M. M., Snyder S. H. (2009). Signaling by gasotransmitters. *Science Signaling*.

[126] Schoenfeld M. P., Ansari R. R., Nakao A., Wink D. (2012). A hypothesis on biological protection from space radiation through the use of new therapeutic gases as medical counter measures. *Medical Gas Research*.

[127] Choi A. M. K., Otterbein L. E. (2002). Emerging role of carbon monoxide in physiologic and pathophysiologic states. *Antioxidants and Redox Signaling*.

[128] Wang R. (2003). The gasotransmitter role of hydrogen sulfide. *Antioxidants and Redox Signaling*.

[129] Rodriguez F., Lamon B. D., Gong W., Kemp R., Nasjletti A. (2004). Nitric oxide synthesis inhibition promotes renal production of carbon monoxide. *Hypertension*.

[130] Jahn N., Lamberts R. R., Busch C. J. (2015). Inhaled carbon monoxide protects time-dependently from loss of hypoxic pulmonary vasoconstriction in endotoxemic mice. *Respiratory Research*.

[131] Kimura H., Shibuya N., Kimura Y. (2012). Hydrogen sulfide is a signaling molecule and a cytoprotectant. *Antioxidants and Redox Signaling*.

[132] Bir S. C., Kolluru G. K., McCarthy P. (2012). Hydrogen sulfide stimulates ischemic vascular remodeling through nitric oxide synthase and nitrite reduction activity regulating hypoxia-inducible factor-1*α* and vascular endothelial growth factor-dependent angiogenesis. *The Journal of the American Heart Association*.

[133] Donnarumma E., Ali M. J., Rushing A. M. (2016). Zofenopril protects against myocardial ischemia-reperfusion injury by increasing nitric oxide and hydrogen sulfide bioavailability. *Journal of the American Heart Association*.

[134] Westlin W., Mullane K. (1988). Does captopril attenuate reperfusion-induced myocardial dysfunction by scavenging free radicals?. *Circulation*.

[135] Borghi C., Ambrosioni E. (2003). Double-blind comparison between zofenopril and lisinopril in patients with acute myocardial infarction: Results of the Survival of Myocardial Infarction Long-term Evaluation-2 (SMILE-2) Study. *American Heart Journal*.

[136] Bayard V., Chamorro F., Motta J., Hollenberg N. K. (2007). Does flavanol intake influence mortality from nitric oxide-dependent processes? Ischemic heart disease, stroke, diabetes mellitus, and cancer in Panama. *International Journal of Medical Sciences*.

[137] Buijsse B., Feskens E. J. M., Kok F. J., Kromhout D. (2006). Cocoa intake, blood pressure, and cardiovascular mortality: The Zutphen Elderly Study. *Archives of Internal Medicine*.

[138] D’Alessandro A., De Pergola G. (2015). Mediterranean diet and cardiovascular disease: a critical evaluation of a priori dietary indexes. *Nutrients*.

[139] Estruch R., Martínez-González M. A., Corella D. (2006). Effects of a mediterranean-style diet on cardiovascular risk factors: a randomized trial. *Annals of Internal Medicine*.

[140] Arts I. C. W., Hollman P. C. H., Feskens E. J. M., Bueno de Mesquita H. B., Kromhout D. (2001). Catechin intake might explain the inverse relation between tea consumption and ischemic heart disease: The Zutphen Elderly Study. *American Journal of Clinical Nutrition*.

[141] Buijsse B., Weikert C., Drogan D., Bergmann M., Boeing H. (2010). Chocolate consumption in relation to blood pressure and risk of cardiovascular disease in German adults. *European Heart Journal*.

[142] Liu B.-L., Zhang X., Zhang W., Zhen H.-N. (2007). New enlightenment of French paradox: resveratrol's potential for cancer chemoprevention and anti-cancer therapy. *Cancer Biology and Therapy*.

[143] Upadhyay S., Dixit M. (2015). Role of polyphenols and other phytochemicals on molecular signaling. *Oxidative Medicine and Cellular Longevity*.

[144] Kreft S., Knapp M., Kreft I. (1999). Extraction of rutin from buckwheat (*Fagopyrum esculentum* moench) seeds and determination by capillary electrophoresis. *Journal of Agricultural and Food Chemistry*.

[145] Stewart A. J., Bozonnet S., Mullen W., Jenkins G. I., Lean M. E. J., Crozier A. (2000). Occurrence of flavonols in tomatoes and tomato-based products. *Journal of Agricultural and Food Chemistry*.

[146] Reinli K., Block G. (1996). Phytoestrogen content of foods—a compendium of literature values. *Nutrition and Cancer*.

[147] López M., Martínez F., Del Valle C., Orte C., Miró M. (2001). Analysis of phenolic constituents of biological interest in red wines by high-performance liquid chromatography. *Journal of Chromatography A*.

[148] López-Lázaro M. (2009). Distribution and biological activities of the flavonoid luteolin. *Mini-Reviews in Medicinal Chemistry*.

[149] Gupta K. K., Taneja S. C., Dhar K. L., Atal C. K. (1983). Flavonoids of andrographis paniculata. *Phytochemistry*.

[150] Khan N., Afaq F., Syed D. N., Mukhtar H. (2008). Fisetin, a novel dietary flavonoid, causes apoptosis and cell cycle arrest in human prostate cancer LNCaP cells. *Carcinogenesis*.

[151] Yang P.-M., Tseng H.-H., Peng C.-W., Chen W.-S., Chiu S.-J. (2012). Dietary flavonoid fisetin targets caspase-3-deficient human breast cancer MCF-7 cells by induction of caspase-7-associated apoptosis and inhibition of autophagy. *International Journal of Oncology*.

[152] Heim K. E., Tagliaferro A. R., Bobilya D. J. (2002). Flavonoid antioxidants: chemistry, metabolism and structure-activity relationships. *Journal of Nutritional Biochemistry*.

[153] Lamson D. W., Brignall M. S. (2000). Antioxidants and cancer III: quercetin. *Alternative Medicine Review*.

[154] Hirata H., Ueno K., Nakajima K. (2013). Genistein downregulates onco-miR-1260b and inhibits Wnt-signalling in renal cancer cells. *British Journal of Cancer*.

[155] Mahmoud A. M., Yang W., Bosland M. C. (2014). Soy isoflavones and prostate cancer: a review of molecular mechanisms. *The Journal of Steroid Biochemistry and Molecular Biology*.

[156] Shen T., Wang X.-N., Lou H.-X. (2009). Natural stilbenes: an overview. *Natural Product Reports*.

[157] Wang Z., Zou J., Cao K., Hsieh T.-C., Huang Y., Wu J. M. (2005). Dealcoholized red wine containing known amounts of resveratrol suppresses atherosclerosis in hypercholesterolemic rabbits without affecting plasma lipid levels. *International Journal of Molecular Medicine*.

[158] Hurst W. J., Glinski J. A., Miller K. B., Apgar J., Davey M. H., Stuart D. A. (2008). Survey of the trans-resveratrol and trans-piceid content of cocoa-containing and chocolate products. *Journal of Agricultural and Food Chemistry*.

[159] Salem S., Shafique A., Dore S. (2006). *Protective Effects of Resveratrol in Age-Related Neurodegenerative Diseases and Gene Regulatory Action*.

[160] Fiorentino A., D'Abrosca B., Pacifico S., Cefarelli G., Uzzo P., Monaco P. (2007). Natural dibenzoxazepinones from leaves of *Carex distachya*: structural elucidation and radical scavenging activity. *Bioorganic and Medicinal Chemistry Letters*.

[161] Shankar S., Chen Q., Siddiqui I., Sarva K., Srivastava R. K. (2014). Sensitization of TRAIL-resistant LNCaP cells by resveratrol (3, 4′, 5 tri-hydroxystilbene): molecular mechanisms and therapeutic potential. *Journal of Molecular Signaling*.

[162] van Ginkel P. R., Yan M. B., Bhattacharya S., Polans A. S., Kenealey J. D. (2015). Natural products induce a G protein-mediated calcium pathway activating p53 in cancer cells. *Toxicology and Applied Pharmacology*.

[163] McCormack D., McFadden D. (2013). A review of pterostilbene antioxidant activity and disease modification. *Oxidative Medicine and Cellular Longevity*.

[164] Mena S., Rodríguez M. L., Ponsoda X., Estrela J. M., Jäättela M., Ortega A. L. (2012). Pterostilbene-induced tumor cytotoxicity: a lysosomal membrane permeabilization-dependent mechanism. *PLoS ONE*.

[165] Banerjee M., Singh P., Panda D. (2010). Curcumin suppresses the dynamic instability of microtubules, activates the mitotic checkpoint and induces apoptosis in MCF-7 cells. *The FEBS Journal*.

[166] Balasubramanian S., Eckert R. L. (2007). Curcumin suppresses AP1 transcription factor-dependent differentiation and activates apoptosis in human epidermal keratinocytes. *The Journal of Biological Chemistry*.

[167] Jayaprakasha G. K., Jaganmohan Rao L., Sakariah K. K. (2006). Antioxidant activities of curcumin, demethoxycurcumin and bisdemethoxycurcumin. *Food Chemistry*.

[168] Carluccio M. A., Siculella L., Ancora M. A. (2003). Olive oil and red wine antioxidant polyphenols inhibit endothelial activation: antiatherogenic properties of Mediterranean diet phytochemicals. *Arteriosclerosis, Thrombosis, and Vascular Biology*.

[169] Khayyal M. T., El-Ghazaly M. A., Abdallah D. M., Nassar N. N., Okpanyi S. N., Kreuter M.-H. (2002). Blood pressure lowering effect of an olive leaf extract (*Olea europaea*) in L-NAME induced hypertension in rats. *Arzneimittel-Forschung/Drug Research*.

[170] Jemai H., Feki A. E. L., Sayadi S. (2009). Antidiabetic and antioxidant effects of hydroxytyrosol and oleuropein from olive leaves in alloxan-diabetic rats. *Journal of Agricultural and Food Chemistry*.

[171] Bulotta S., Corradino R., Celano M. (2011). Antiproliferative and antioxidant effects on breast cancer cells of oleuropein and its semisynthetic peracetylated derivatives. *Food Chemistry*.

[172] Manna C., D'Angelo S., Migliardi V. (2002). Protective effect of the phenolic fraction from virgin olive oils against oxidative stress in human cells. *Journal of Agricultural and Food Chemistry*.

[173] Owen R. W., Giacosa A., Hull W. E. (2000). Olive-oil consumption and health: the possible role of antioxidants. *The Lancet Oncology*.

[174] Granados-Principal S., Quiles J. L., Ramirez-Tortosa C. L., Sanchez-Rovira P., Ramirez-Tortosa M. C. (2010). Hydroxytyrosol: from laboratory investigations to future clinical trials. *Nutrition Reviews*.

[175] Conti V., Izzo V., Corbi G. (2016). Antioxidant supplementation in the treatment of aging-associated diseases. *Frontiers in Pharmacology*.

[176] Karim M., Mccormick K., Kappagoda C. T. (2000). Chocolate: modern science investigates an ancient medicine: effects of cocoa extracts on endothelium-dependent relaxation. *Journal of Nutrition*.

[177] Taubert D., Berkels R., Klaus W., Roesen R. (2002). Nitric oxide formation and corresponding relaxation of porcine coronary arteries induced by plant phenols: essential structural features. *Journal of Cardiovascular Pharmacology*.

[178] Lorenz M., Wessler S., Follmann E. (2004). A constituent of green tea, epigallocatechin-3-gallate, activates endothelial nitric oxide synthase by a phosphatidylinositol-3-OH-kinase-, cAMP-dependent protein kinase-, and Akt-dependent pathway and leads to endothelial-dependent vasorelaxation. *The Journal of Biological Chemistry*.

[179] Kim J.-A., Formoso G., Li Y. (2007). Epigallocatechin gallate, a green tea polyphenol, mediates NO-dependent vasodilation using signaling pathways in vascular endothelium requiring reactive oxygen species and fyn. *The Journal of Biological Chemistry*.

[180] Ihm S.-H., Lee J.-O., Kim S.-J. (2009). Catechin prevents endothelial dysfunction in the prediabetic stage of OLETF rats by reducing vascular NADPH oxidase activity and expression. *Atherosclerosis*.

[181] Duffy S. J., Vita J. A., Holbrook M., Swerdloff P. L., Keaney J. F. (2001). Effect of acute and chronic tea consumption on platelet aggregation in patients with coronary artery disease. *Arteriosclerosis, Thrombosis, and Vascular Biology*.

[182] Widlansky M. E., Hamburg N. M., Anter E. (2007). Acute EGCG supplementation reverses endothelial dysfunction in patients with coronary artery disease. *Journal of the American College of Nutrition*.

[183] Anter E., Thomas S. R., Schulz E., Shapira O. M., Vita J. A., Keaney J. F. (2004). Activation of endothelial nitric-oxide synthase by the p38 MAPK in response to black tea polyphenols. *The Journal of Biological Chemistry*.

[184] Piao M. J. I., Yoo E. S. O., Koh Y. S. A. (2011). Antioxidant effects of the ethanol extract from flower of *Camellia japonica* via scavenging of reactive oxygen species and induction of antioxidant enzymes. *International Journal of Molecular Sciences*.

[185] Park S.-H., Shim B.-S., Yoon J.-S. (2016). Vascular protective effect of an ethanol extract of *Camellia japonica* fruit: endothelium-dependent relaxation of coronary artery and reduction of smooth muscle cell migration. *Oxidative Medicine and Cellular Longevity*.

[186] Chen L.-G., Liu Y.-C., Hsieh C.-W., Liao B.-C., Wung B.-S. (2008). Tannin 1-*α*-*O*-galloylpunicalagin induces the calcium-dependent activation of endothelial nitric-oxide synthase via the phosphatidylinositol 3-kinase/Akt pathway in endothelial cells. *Molecular Nutrition and Food Research*.

[187] Appeldoorn M. M., Venema D. P., Peters T. H. F. (2009). Some phenolic compounds increase the nitric oxide level in endothelial cells in vitro. *Journal of Agricultural and Food Chemistry*.

[188] Duarte J., Pérez-Palencia R., Vargas F. (2001). Antihypertensive effects of the flavonoid quercetin in spontaneously hypertensive rats. *British Journal of Pharmacology*.

[189] Galisteo M., García-Saura M. F., Jiménez R. (2004). Effects of quercetin treatment on vascular function in deoxycorticosterone acetate-salt hypertensive rats. Comparative study with verapamil. *Planta Medica*.

[190] Galisteo M., García-Saura M. F., Jiménez R. (2004). Effects of chronic quercetin treatment on antioxidant defence system and oxidative status of deoxycorticosterone acetate-salt-hypertensive rats. *Molecular and Cellular Biochemistry*.

[191] Duarte J., Jiménez R., O'Valle F. (2002). Protective effects of the flavonoid quercetin in chronic nitric oxide deficient rats. *Journal of Hypertension*.

[192] Zhang Y., Huang C., Liu S. (2015). Effects of quercetin on intracavernous pressure and expression of nitrogen synthase isoforms in arterial erectile dysfunction rat model. *International Journal of Clinical and Experimental Medicine*.

[193] Perez-Vizcaino F., Duarte J., Jimenez R., Santos-Buelga C., Osuna A. (2009). Antihypertensive effects of the flavonoid quercetin. *Pharmacological Reports*.

[194] Sánchez M., Galisteo M., Vera R. (2006). Quercetin downregulates NADPH oxidase, increases eNOS activity and prevents endothelial dysfunction in spontaneously hypertensive rats. *Journal of Hypertension*.

[195] Jin B.-H., Qian L.-B., Chen S. (2009). Apigenin protects endothelium-dependent relaxation of rat aorta against oxidative stress. *European Journal of Pharmacology*.

[196] Xu P.-H., Long Y., Dai F., Liu Z.-L. (2007). The relaxant effect of curcumin on porcine coronary arterial ring segments. *Vascular Pharmacology*.

[197] Li Y., Ying C., Zuo X. (2009). Green tea polyphenols down-regulate caveolin-1 expression via ERK1/2 and p38MAPK in endothelial cells. *Journal of Nutritional Biochemistry*.

[198] Vera R., Galisteo M., Villar I. C. (2005). Soy isoflavones improve endothelial function in spontaneously hypertensive rats in an estrogen-independent manner: role of nitric-oxide synthase, superoxide, and cyclooxygenase metabolites. *Journal of Pharmacology and Experimental Therapeutics*.

[199] Sobey C. G., Weiler J. M., Boujaoude M., Woodman O. L. (2004). Effect of short-term phytoestrogen treatment in male rats on nitric oxide-mediated responses of carotid and cerebral arteries: comparison with 17*β*-estradiol. *Journal of Pharmacology and Experimental Therapeutics*.

[200] Hwang J., Wang J., Morazzoni P., Hodis H. N., Sevanian A. (2003). The phytoestrogen equol increases nitric oxide availability by inhibiting superoxide production: an antioxidant mechanism for cell-mediated LDL modification. *Free Radical Biology and Medicine*.

[201] Yamamoto M., Suzuki A., Hase T. (2008). Short-term effects of glucosyl hesperidin and hesperetin on blood pressure and vascular endothelial function in spontaneously hypertensive rats. *Journal of Nutritional Science and Vitaminology*.

[202] Yamamoto M., Suzuki A., Jokura H., Yamamoto N., Hase T. (2008). Glucosyl hesperidin prevents endothelial dysfunction and oxidative stress in spontaneously hypertensive rats. *Nutrition*.

[203] Smith P. D. C. (2000). Micronized purified flavonoid fraction and the treatment of chronic venous insufficiency: microcirculatory mechanisms. *Microcirculation*.

[204] Cienfuegos-Jovellanos E., Del Mar Quiñones M., Muguerza B., Moulay L., Miguel M., Aleixandre A. (2009). Antihypertensive effect of a polyphenol-rich cocoa powder industrially processed to preserve the original flavonoids of the cocoa beans. *Journal of Agricultural and Food Chemistry*.

[205] Taubert D., Roesen R., Lehmann C., Jung N., Schömig E. (2007). Effects of low habitual cocoa intake on blood pressure and bioactive nitric oxide: a randomized controlled trial. *Journal of the American Medical Association*.

[206] Engler M. B., Engler M. M., Chen C. Y. (2004). Flavonoid-rich dark chocolate improves endothelial function and increases plasma epicatechin concentrations in healthy adults. *Journal of the American College of Nutrition*.

[207] Schroeter H., Heiss C., Balzer J. (2006). (−)-Epicatechin mediates beneficial effects of flavanol-rich cocoa on vascular function in humans. *Proceedings of the National Academy of Sciences of the United States of America*.

[208] Fisher N. D. L., Hughes M., Gerhard-Herman M., Hollenberg N. K. (2003). Flavanol-rich cocoa induces nitric-oxide-dependent vasodilation in healthy humans. *Journal of Hypertension*.

[209] Medina-Remón A., Tresserra-Rimbau A., Pons A. (2015). Effects of total dietary polyphenols on plasma nitric oxide and blood pressure in a high cardiovascular risk cohort. The PREDIMED Randomized Trial. *Nutrition, Metabolism and Cardiovascular Diseases*.

[210] Fitzpatrick D. F., Hirschfield S. L., Coffey R. G. (1993). Endothelium-dependent vasorelaxing activity of wine and other grape products. *American Journal of Physiology—Heart and Circulatory Physiology*.

[211] Mendes A., Desgranges C., Chèze C., Vercauteren J., Freslon J.-L. (2003). Vasorelaxant effects of grape polyphenols in rat isolated aorta. Possible involvement of a purinergic pathway. *Fundamental and Clinical Pharmacology*.

[212] Andriambeloson E., Kleschyov A. L., Muller B., Beretz A., Stoclet J. C., Andriantsitohaina R. (1997). Nitric oxide production and endothelium-dependent vasorelaxation induced by wine polyphenols in rat aorta. *British Journal of Pharmacology*.

[213] Leikert J. F., Räthel T. R., Wohlfart P., Cheynier V., Vollmar A. M., Dirsch V. M. (2002). Red wine polyphenols enhance endothelial nitric oxide synthase expression and subsequent nitric oxide release from endothelial cells. *Circulation*.

[214] Martin S., Andriambeloson E., Takeda K., Andriantsitohaina R. (2002). Red wine polyphenols increase calcium in bovine aortic endothelial cells: a basis to elucidate signalling pathways leading to nitric oxide production. *British Journal of Pharmacology*.

[215] Edirisinghe I., Burton-Freeman B., Kappagoda C. T. (2008). Mechanism of the endothelium-dependent relaxation evoked by a grape seed extract. *Clinical Science*.

[216] Madeira S. V. F., Auger C., Anselm E. (2009). eNOS activation induced by a polyphenol-rich grape skin extract in porcine coronary arteries. *Journal of Vascular Research*.

[217] Ndiaye M., Chataigneau M., Lobysheva I., Chataigneau T., Schini-Kerth V. B. (2005). Red wine polyphenol-induced, endothelium-dependent NO-mediated relaxation is due to the redox-sensitive PI3-kinase/Akt-dependent phosphorylation of endothelial NO-synthase in the isolated porcine coronary artery. *FASEB Journal*.

[218] Zenebe W., Pecháňová O., Andriantsitohaina R. (2003). Red wine polyphenols induce vasorelaxation by increased nitric oxide bioactivity. *Physiological Research*.

[219] Benito S., Lopez D., Sáiz M. P. (2002). A flavonoid-rich diet increases nitric oxide production in rat aorta. *British Journal of Pharmacology*.

[220] Bernátová I., Pechánová O., Babál P., Kyselá S., Stvrtina S., Andriantsitohaina R. (2002). Wine polyphenols improve cardiovascular remodeling and vascular function in NO-deficient hypertension. *American Journal of Physiology-Heart and Circulatory Physiology*.

[221] Jiménez R., López-Sepúlveda R., Kadmiri M. (2007). Polyphenols restore endothelial function in DOCA-salt hypertension: role of endothelin-1 and NADPH oxidase. *Free Radical Biology and Medicine*.

[222] Sarr M., Chataigneau M., Martins S. (2006). Red wine polyphenols prevent angiotensin II-induced hypertension and endothelial dysfunction in rats: role of NADPH oxidase. *Cardiovascular Research*.

[223] Walter A., Etienne-Selloum N., Brasse D. (2009). Red wine polyphenols prevent acceleration of neovascularization by angiotensin II in the ischemic rat hindlimb. *Journal of Pharmacology and Experimental Therapeutics*.

[224] Napoli C., Balestrieri M. L., Sica V. (2008). Beneficial effects of low doses of red wine consumption on perturbed shear stress-induced atherogenesis. *Heart and Vessels*.

[225] Magrone T., Tafaro A., Jirillo F. (2007). Red wine consumption and prevention of atherosclerosis: an in vitro model using human peripheral blood mononuclear cells. *Current Pharmaceutical Design*.

[226] Freedman J. E., Parker C., Li L. (2001). Select flavonoids and whole juice from purple grapes inhibit platelet function and enhance nitric oxide release. *Circulation*.

[227] Keevil J. G., Osman H. E., Reed J. D., Folts J. D. (2000). Grape juice, but not orange juice or grapefruit juice, inhibits human platelet aggregation. *Journal of Nutrition*.

[228] Huang P.-H., Chen Y.-H., Tsai H.-Y. (2010). Intake of red wine increases the number and functional capacity of circulating endothelial progenitor cells by enhancing nitric oxide bioavailability. *Arteriosclerosis, Thrombosis, and Vascular Biology*.

[229] Chiva-Blanch G., Urpi-Sarda M., Ros E. (2012). Dealcoholized red wine decreases systolic and diastolic blood pressure and increases plasma nitric oxide: short communication. *Circulation Research*.

[230] Karatzi K., Papamichael C., Karatzis E. (2007). Acute smoking induces endothelial dysfunction in healthy smokers. Is this reversible by red wine's antioxidant constituents?. *Journal of the American College of Nutrition*.

[231] Carrizzo A., Forte M., Lembo M., Formisano L., Puca A. A., Vecchione C. (2014). Rac-1 as a new therapeutic target in cerebro-and cardio-vascular diseases. *Current Drug Targets*.

[232] Prasad K. (2012). Resveratrol, wine, and atherosclerosis. *The International Journal of Angiology*.

[233] Jang M., Cai L., Udeani G. O. (1997). Cancer chemopreventive activity of resveratrol, a natural product derived from grapes. *Science*.

[234] Timmers S., Konings E., Bilet L. (2011). Calorie restriction-like effects of 30 days of resveratrol supplementation on energy metabolism and metabolic profile in obese humans. *Cell Metabolism*.

[235] Li H., Xia N., Förstermann U. (2012). Cardiovascular effects and molecular targets of resveratrol. *Nitric Oxide-Biology and Chemistry*.

[236] Norata G. D., Marchesi P., Passamonti S., Pirillo A., Violi F., Catapano A. L. (2007). Anti-inflammatory and anti-atherogenic effects of cathechin, caffeic acid and trans-resveratrol in apolipoprotein E deficient mice. *Atherosclerosis*.

[237] Conti V., Forte M., Corbi G. (2015). Sirtuins: a possible clinical implication in cardio- and cerebro- vascular systems. *Current Drug Targets*.

[238] Xia N., Strand S., Schlufter F. (2013). Role of SIRT1 and FOXO factors in eNOS transcriptional activation by resveratrol. *Nitric Oxide-Biology and Chemistry*.

[239] Wallerath T., Deckert G., Ternes T. (2002). Resveratrol, a polyphenolic phytoalexin present in red wine, enhances expression and activity of endothelial nitric oxide synthase. *Circulation*.

[240] Xia N., Daiber A., Habermeier A. (2010). Resveratrol reverses endothelial nitric-oxide synthase uncoupling in apolipoprotein E knockout mice. *Journal of Pharmacology and Experimental Therapeutics*.

[241] Zou J.-G., Wang Z.-R., Huang Y.-Z., Cao K.-J., Wu J. M. (2003). Effect of red wine and wine polyphenol resveratrol on endothelial function in hypercholesterolemic rabbits. *International Journal of Molecular Medicine*.

[242] Xu Q., Hao X., Yang Q., Si L. (2009). Resveratrol prevents hyperglycemia-induced endothelial dysfunction via activation of adenosine monophosphate-activated protein kinase. *Biochemical and Biophysical Research Communications*.

[243] Zhang H., Zhang J., Ungvari Z., Zhang C. (2009). Resveratrol improves endothelial function: role of TNF*α* and vascular oxidative stress. *Arteriosclerosis, Thrombosis, and Vascular Biology*.

[244] Wang N., Ko S.-H., Chai W. (2011). Resveratrol recruits rat muscle microvasculature via a nitric oxide-dependent mechanism that is blocked by TNF*α*. *American Journal of Physiology-Endocrinology and Metabolism*.

[245] Rivera L., Morón R., Zarzuelo A., Galisteo M. (2009). Long-term resveratrol administration reduces metabolic disturbances and lowers blood pressure in obese Zucker rats. *Biochemical Pharmacology*.

[246] Cheng P.-W., Ho W.-Y., Su Y.-T. (2014). Resveratrol decreases fructose-induced oxidative stress, mediated by NADPH oxidase via an AMPK-dependent mechanism. *British Journal of Pharmacology*.

[247] Penumathsa S. V., Thirunavukkarasu M., Zhan L. (2008). Resveratrol enhances GLUT-4 translocation to the caveolar lipid raft fractions through AMPK/Akt/eNOS signalling pathway in diabetic myocardium. *Journal of Cellular and Molecular Medicine*.

[248] Penumathsa S. V., Koneru S., Samuel S. M. (2008). Strategic targets to induce neovascularization by resveratrol in hypercholesterolemic rat myocardium: role of caveolin-1, endothelial nitric oxide synthase, hemeoxygenase-1, and vascular endothelial growth factor. *Free Radical Biology and Medicine*.

[249] Hattori R., Otani H., Maulik N., Das D. K. (2002). Pharmacological preconditioning with resveratrol: Role of nitric oxide. *American Journal of Physiology—Heart and Circulatory Physiology*.

[250] Dolinsky V. W., Chakrabarti S., Pereira T. J. (2013). Resveratrol prevents hypertension and cardiac hypertrophy in hypertensive rats and mice. *Biochimica et Biophysica Acta-Molecular Basis of Disease*.

[251] Bhatt S. R., Lokhandwala M. F., Banday A. A. (2011). Resveratrol prevents endothelial nitric oxide synthase uncoupling and attenuates development of hypertension in spontaneously hypertensive rats. *European Journal of Pharmacology*.

[252] Gresele P., Pignatelli P., Guglielmini G. (2008). Resveratrol, at concentrations attainable with moderate wine consumption, stimulates human platelet nitric oxide production. *Journal of Nutrition*.

[253] Gu J., Wang C. Q., Fan H. H. (2006). Effects of resveratrol on endothelial progenitor cells and their contributions to reendothelialization in intima-injured rats. *Journal of Cardiovascular Pharmacology*.

[254] Carrizzo A., Puca A., Damato A. (2013). Resveratrol improves vascular function in patients with hypertension and dyslipidemia by modulating NO metabolism. *Hypertension*.

[255] Clark D., Tuor U. I., Thompson R. (2012). Protection against recurrent stroke with resveratrol: endothelial protection. *PLoS ONE*.

[256] Tsai S.-K., Hung L.-M., Fu Y.-T. (2007). Resveratrol neuroprotective effects during focal cerebral ischemia injury via nitric oxide mechanism in rats. *Journal of Vascular Surgery*.

[257] Chen J., Lin H., Hu M. (2003). Metabolism of flavonoids via enteric recycling: role of intestinal disposition. *Journal of Pharmacology and Experimental Therapeutics*.

[258] Smoliga J. M., Vang O., Baur J. A. (2012). Challenges of translating basic research into therapeutics: resveratrol as an example. *Journals of Gerontology—Series A Biological Sciences and Medical Sciences*.

[259] Khushnud T., Mousa S. A. (2013). Potential role of naturally derived polyphenols and their nanotechnology delivery in cancer. *Molecular Biotechnology*.

[260] Mayol L., Serri C., Menale C. (2015). Curcumin loaded PLGA-poloxamer blend nanoparticles induce cell cycle arrest in mesothelioma cells. *European Journal of Pharmaceutics and Biopharmaceutics*.

